# Flexible synthesis of cationic peptide–porphyrin derivatives for light-triggered drug delivery and photodynamic therapy†
†Electronic supplementary information (ESI) available: Copies of ^1^H and ^13^C spectra, HPLC traces, UV and fluorescence spectra. See DOI: 10.1039/c6ob02135b



**DOI:** 10.1039/c6ob02135b

**Published:** 2016-11-14

**Authors:** R. Dondi, E. Yaghini, K. M. Tewari, L. Wang, F. Giuntini, M. Loizidou, A. J. MacRobert, I. M. Eggleston

**Affiliations:** a Department of Pharmacy and Pharmacology , University of Bath , Bath BA2 7AY , UK . Email: ie203@bath.ac.uk; b UCL Division of Surgery and Interventional Science , University College London , Royal Free Campus , Rowland Hill Street , London NW3 2PF , UK; c School of Pharmaceutical Sciences , Shandong University , Jinan , China

## Abstract

Efficient syntheses of cell-penetrating peptide–porphyrin conjugates are described using a variety of bioconjugation chemistries. This provides a flexible means to convert essentially hydrophobic tetrapyrolle photosensitisers into amphiphilic derivatives which are well-suited for use in light-triggered drug delivery by photochemical internalisation (PCI) and targeted photodynamic therapy (PDT).

## Abstract 2

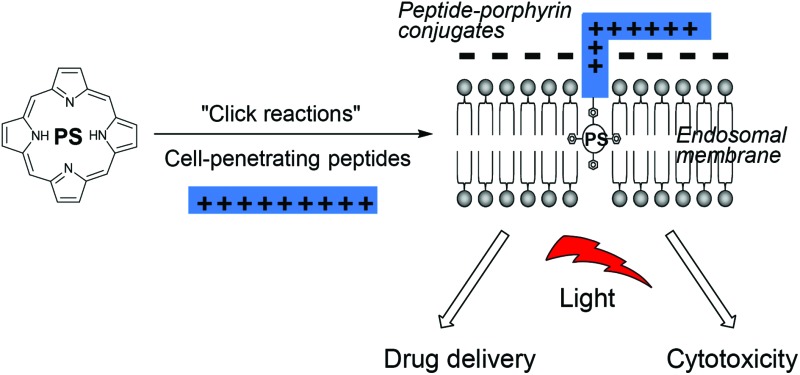
Amphiphilic cell-penetrating peptide–porphyrin conjugates have been developed for application in light-based therapeutic techniques.

## Introduction

Photodynamic therapy (PDT) is a light-based therapeutic technique that shows great promise for the selective treatment of a variety of cancers and non-malignant conditions.^1^ The principle of PDT is that highly localised destruction of tumours, diseased tissue or pathogenic organisms is achieved with light following the administration of a light-activated photosensitising drug or photosensitiser. This generates various cytotoxic reactive oxygen species such as singlet oxygen that can interact with and damage cellular components, leading to cell death. Many photosensitisers have been developed and have received approval or clinical trials for use in the treatment of cancer,^2^ age-related macular degeneration,^3^ pre-cancerous conditions such as Barret's oesphagus,^4^ and oral sterilisation in dental procedures.^5^ Among these, porphyrins and other tetrapyrrole-related compounds remain the broadest class of compound under study for PDT and other light-based therapies.^6^ The effectiveness of PDT in a particular application depends upon the wavelength at which the photosensitiser may be activated, and this has led to the development of many modified tetrapyrroles that show enhanced absorption at the red end of the spectrum where absorption by tissue is relatively weak, thus allowing a photodynamic effect at a greater depth *in vivo.* ^6,7^ Another important consideration in the development of novel photosensitisers is their efficient delivery to the target site, in particular achieving selective delivery of photosensitisers to tumours compared to normal tissues. In this context, in recent years there has been considerable interest in the development of peptide and protein-targeted photosensitisers as a means of improving the pharmacokinetic properties, solubility and tissue specificity of various otherwise hydrophobic derivatives. Conjugation of photosensitisers to antibodies and a range of synthetic peptides has been explored and has been found to provide significant enhancements in both the efficiency and selectivity of cellular uptake of photosensitisers for PDT in a range of cancer models.^8^ A key innovation in the development of such targeted photosensitisers has been the application of biorthogonal ligation techniques^9^ which facilitate the efficient attachment of a variety of photosensitisers to unprotected, multifunctional peptides in solution.

Cell-penetrating peptides (CPPs) are an intensely researched class of biocompatible carriers which have great potential for targeted drug delivery.^10–12^ Such molecules, of either natural or synthetic origin typically consist of 8–30 amino acid residues and possess the ability to translocate across biological membranes and transport diverse molecular cargoes, either covalently or non-covalently attached, which would otherwise be poorly internalised. The conjugation of tetrapyrrole-based photosensitisers and other derivatives to CPPs has indeed been shown to provide an attractive means for enhancing photosensitiser delivery for both PDT of cancer^13–15^ and in antimicrobial PDT applications.^16,17^ Importantly, CPP-conjugation also offers a means to control the sub-cellular localisation of a particular photosensitiser in eukaryotic systems to potentially maximise therapeutic effect.^10,14^

We recently demonstrated that the conjugation of a porphyrin derivative to a cationic CPP is an effective way of generating a novel water-soluble amphiphilic photosensitiser suitable for light-triggered drug delivery by photochemical internalisation (PCI).^18^ PCI is a novel technology for effecting the light-activated intracellular release of biologically active molecules at specific sites in living tissue, often where conventional methods of drug administration prove unsuccessful.^19,20^ The effectiveness of various macromolecular therapeutic agents can often be severely impaired by their limited ability to reach their intracellular targets due to sequestration in endosomes and lysosomes after uptake by endocytic mechanisms. PCI provides a highly effective means to selectively trigger endosomal escape and intracellular relocalisation of entrapped drugs, by applying the basic principle of the PDT approach. In PCI, a sub-lethal light dose is applied to a photosensitiser that localises in endo/lysosomal membranes, which is sufficient to cause partial rupture of these intracellular organelles (mediated by singlet oxygen). This allows the entrapped drugs to escape but does not compromise the viability of the cells themselves.^20^ Recent clinical studies have demonstrated both the feasibility and safety of PCI for the treatment of advanced and recurrent solid malignancies.^21^

**Fig. 1:**
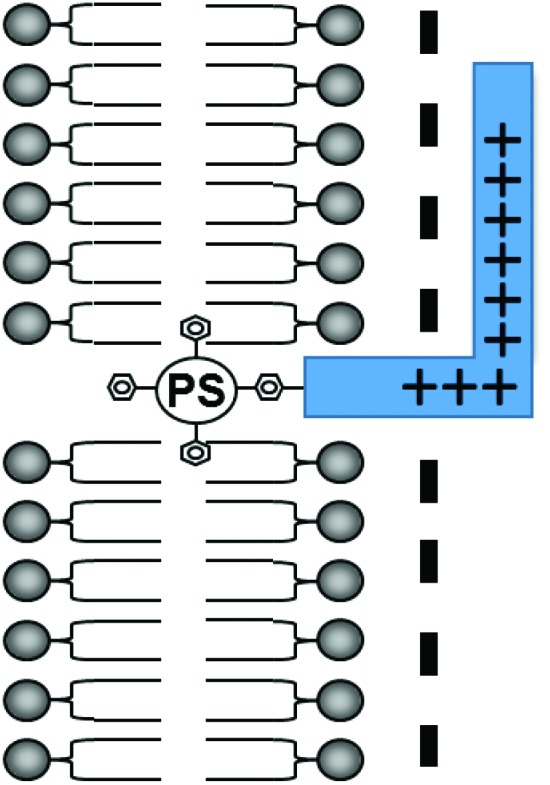
Amphiphilic CPP-targeted photosensitiser. The hydrophobic photosensitiser unit localises in the lipid bilayer of endosomal/lysosomal vesicles while the cationic peptide carrier resides in the fluid phase.

A critical requirement in PCI is that the photosensitiser used must possess features that make it localise in the same intracellular vesicles (lysosomes, endosomes) as the administered drug, *i.e.* they must be *lysosomotropic* and therefore amphiphilic.^20^ Many well-known PDT photosensitisers are unsuitable for PCI as they partition non-selectively to other cellular organelles (*e.g.* mitochondria, Golgi apparatus, endoplasmic reticulum), however as shown in Fig. 1, attachment of an otherwise hydrophobic photosensitiser derivative to a cationic CPP may transform it into an amphiphilic compound that is both water-soluble and amenable to cellular uptake by endocytosis.^18^ The hydrophobic porphyrin macrocycle then has the potential to localise in the lipid bilayer of the endosomal membranes, for selective oxidative damage,^22,23^ with the hydrophilic peptide in the fluid phase.^18^ These properties are ideally suited for PCI (see above).

The application of CPP-targeted photosensitisers for PCI and PDT depends on the availability of reliable synthetic protocols to derivatise typical cationic CPPs in a site-specific fashion^24^ with easily accessible functionalised photosensitisers of maximum clinical potential.^25^ The aim of this study was to investigate the potential of a number of bioconjugation reactions for the efficient modular preparation of CPP–porphyrin conjugates, as a general strategy to convert classical PDT agents into amphiphilic conjugates suitable for targeted PDT and PCI.

## Results and discussion

### Photosensitiser synthesis

**Scheme 1:**
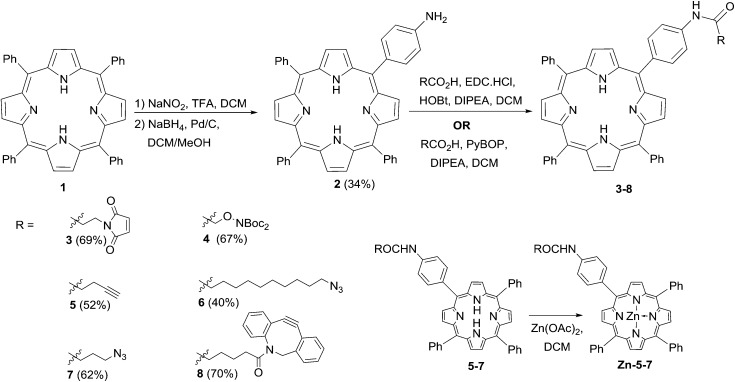
Synthesis of ligatable porphyrin derivatives.

The preparation of the ligatable photosensitisers used in this study is outlined in Scheme 1. As in previous studies by us, *meso*-tetrakistetraphenylporphyrin **1** provided the template for these compounds, and was transformed to the mono-amino derivative **2** by a modification of the method of Luguya *et al.* ^26^ Treatment of **1** with sodium nitrite and trifluoroacetic acid under the reported conditions (3 min contact time) gave a mixture of mono- and dinitrated porphyrins that were reduced directly with sodium borohydride and palladium on charcoal. **2** was then easily separated from disubstituted products and unchanged **1** in reproducible yields of around 30% over the two steps. The porphyrin scaffold was then elaborated with several functionalities that would permit biorthogonal ligation with a suitably modified CPP component, *via* acylation with a suitable carboxylic acid component using either EDC/HOBt or PyBOP activation.^27^ Compounds **3–8** were obtained in satisfactory yields *via* either activation protocol, and in the cases of **3** and **8** were used directly with their complementary ligatable CPP partners (see below). The azido and alkynyl derivatives **5–7** were further converted into the corresponding zinc complexes by treatment with Zn(OAc)_2_ in DCM in excellent yields, ready for use in copper-catalysed ligation reactions.

### Peptide synthesis

**Scheme 2:**
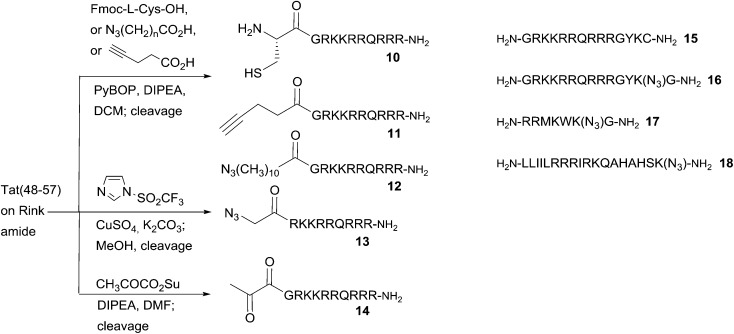
Preparation of N- and C-terminally ligatable CPP derivatives.

All peptides were synthesised by 9-fluorenylmethoxycarbonyl (Fmoc) peptide synthesis on Rink amide resin, using PyBOP activation. Peptides **10–14** were based on the decapeptide CPP sequence derived from the transcriptional activator (Tat) protein from human immunodeficiency virus 1 (HIV-1), commonly known as HIV-1 Tat(48–57),^28^ which provided the template for N-terminally ligatable derivatives in this study. In each case, the final peptide was functionalised on-resin to introduce a complementary reactive group to permit one of the following ligation chemistries with the appropriate porphyrin derivative. Peptides **10–12** were obtained *via* straightforward acylation of the resin-bound peptide with either Fmoc-l-Cys-OH (for **10**), azidoundecanoic acid (for **11**), or pentynoic acid (for **12**), using HATU activation.^27^ For peptide **13**, an N-terminal azido function was introduced by direct diazo transfer upon the resin-bound Tat sequence using the Stick reagent (imidazole-1-sulfonyl azide hydrochloride),^29^ with completion of the reaction being assessed by the Kaiser test,^30^ as for the on-resin acylations to generate **10–12**. In the case of the keto-functionalised derivative **14**, acylation of the resin-bound peptide could only be satisfactorily achieved using the preformed succinimido ester of pyruvic acid.^31^ Other activation methods (DIC, PyBOP, HATU) failed to give desired product, presumably due to the tendency of keto acids such as pyruvate to undergo Claisen-type condensations under strong carboxyl activation conditions.^32^ The C-terminally ligatable peptides **15–18** were obtained directly by standard solid phase peptide synthesis. Cys-containing peptide **15**, was originally reported by Santra *et al.* ^33^ and is also based upon the HIV-1 Tat(48–57) sequence. It provided the template for our prototype CPP-targeted photosensitiser for PCI and applications in antimicrobial PDT.^17,19^ Azidopeptide **16** was derived from **15**, by replacement of the terminal Cys residue with ε-azidolysine^34^ and insertion of a Gly residue at the C-terminus itself, to facilitate loading of the Rink amide resin. Azidopeptides **17** and **18** were obtained in an analogous fashion by respectively the replacement of one Lys residue in the sequence of pVEC, a CPP derived from murine vascular endothelial-cadherin protein, or a fragment of penetratin, a CPP derived from the *Antennapedia* homeoprotein.^12^ The final N-terminally functionalised CPPs **11–14** were obtained in yields of 35–58%, and the C-terminally functionalised derivatives **15–18** in yields of 59–67% after cleavage from the resin and side chain deprotection with TFA/TIS/H_2_O or TFA/TIS/H_2_O/EDT, for Cys-containing peptides **10** and **15** (Scheme 2).

### Ligation reactions

#### Thiol-maleimide and oxime ligation

**Scheme 3:**
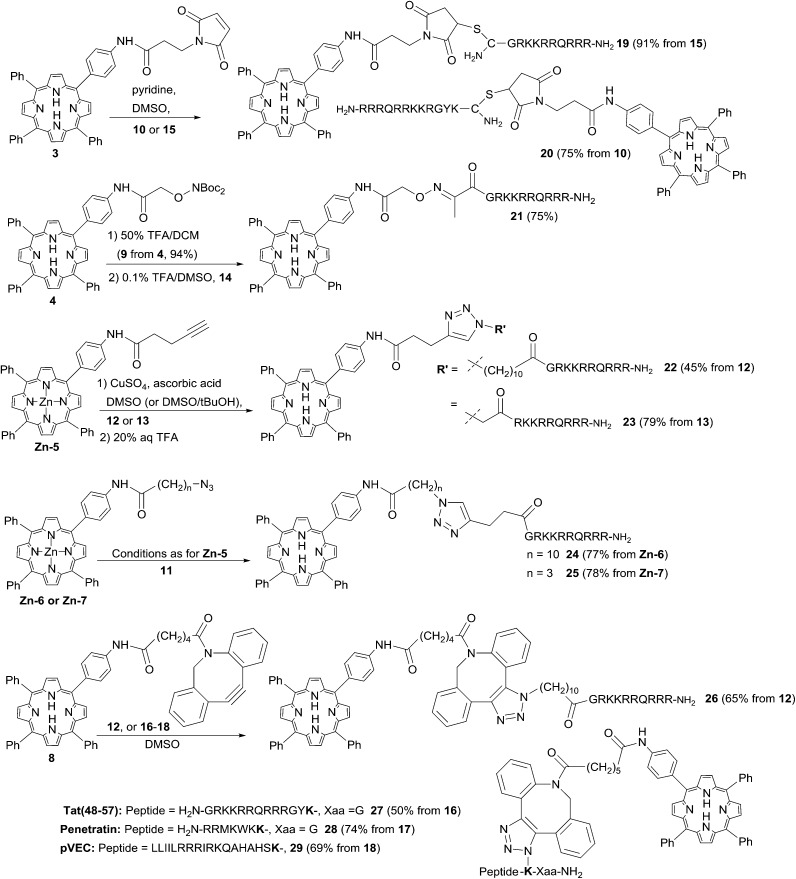
Bioconjugations of functionalised porphyrins to CPP derivatives. The azidolysine-derived residue in conjugates **27–29** is highlighted in bold. Conjugates **27** and **28** contain a spacer residue (Gly) between the azidolysine and the C-terminus.

There are many examples of the ligation of porphyrin-type photosensitisers to multifunctional peptides and proteins with a suitably placed Cys residue *via* conjugate addition to a maleoyl-type or other α,β-unsaturated carbonyl function.^9,35^ Recently, Bryden *et al.* ^36^ have used dibromopyridazinediones as the electrophilic unit as part of a ligation strategy to couple cationic porphyrins to a targeting antibody. In order to compare the efficiency of N- and C-terminal ligation with the cationic Tat sequence, peptides **10** and **15** were reacted with a two-fold excess of porphyrin **31** ^17,37^ in DMSO as shown in Scheme 3. This furnished the desired CPP conjugates **19** and **20** in excellent yields, with no significant difference in the efficiency of N-terminal or C-terminal attachment. This is in contrast to a report by Kitagishi *et al.* ^37^ who observed that an analogous ligation to the N-terminus of an octaarginine derivative proceeded only in very low yield. Indeed, using an alternative polar ligation at the N-terminus of the Tat-CPP unit *via* keto peptide **14** also proved to be highly effective. In this case, the Boc-protected porphyrin **4** was first treated with TFA in DCM to generate aminoxyderivative **9**, which isolated as the free base in 94% yield. The complementary fully deprotected peptide **14** was then treated with a four-fold excess of **9** in 0.1% TFA/DMSO to give the desired oxime conjugate in 75% yield following purification by semi-preparative HPLC. DMSO proved to be the solvent of choice for these ligations as a compromise between the otherwise mutually exclusive solubilities of the highly hydrophobic porphyrins and the unprotected peptides, since preliminary studies with water/PEG systems were found to give very sluggish reactions and variable yields. Notwithstanding this, it was typically possible to greatly simplify the isolation of **19–21** and similar conjugates by precipitation of the crude products or removal of the reaction solvent by isolation on a solid-phase extraction cartridge prior to semi-preparative HPLC.

#### Copper-catalysed azide–alkyne cycloaddition (CuAAC)

Various examples of the ligation of photosensitisers to peptide derivatives using copper-catalysed azide–alkyne cycloaddition (CuAAC) have been described,^38^ however the potential of this approach appears to have been very little exploited with polycationic CPPs as the targeting agent.^39,40^ Although maleimide and oxime ligations proved quite satisfactory for our applications, ligation by copper-catalysed CuAAC offered a number of advantages. As well as generating a triazole-containing linkage that is completely stable under physiological conditions, an additional attractive feature was the possibility to easily introduce one or other of the azide and alkyne functions into either photosensitiser or CPP components, at the N- or C-termini, and with a range of spacer units As a direct comparison for the chemistry of N-terminal attachment, the alkynyl and azido porphyrins **5** and **6** were first combined with the corresponding functionalised Tat peptides **11** and **12** respectively to generate isomeric conjugates **22** and **24** that differ only in the location of the triazole moiety within the linker (Scheme 3). Preliminary tests aimed at devising the optimum reaction conditions confirmed the need for the Zn(ii) complexes of both **5** and **6** to avoid sequestration of the copper catalyst by the macrocycle.^41^ When peptides **11** and **12** were thus reacted with an excess of complexes **Zn-5** and **Zn-6** in the presence of CuSO_4_ and sodium ascorbate in DMSO/H_2_O/*t*BuOH, the N-linked corresponding conjugates were successfully obtained in reproducible yields of 45% and 77% respectively. Conjugates **22** and **24** were isolated by preparative HPLC, and the zinc was easily and quantitatively removed from the porphyrin unit by diluting the reaction mixture with aqueous TFA prior to purification. When the porphyrin component was replaced with azido derivative **Zn-7**, applying the same reaction conditions with Tat peptide **11** also gave the desired conjugate **25** in very good yield (78%); shortening the spacer between the porphyrin unit and the azido function thus appeared to have no serious adverse effect on the cycloaddition reaction. No improvement in yields was observed when the copper source was replaced with a Cu(i) reagent in these reactions (*e.g.* Cu(OTf)),^42^ although the conjugations also proceeded, though generally less efficiently, in the absence of *tert*-butanol as co-solvent. Reaction of alkynyl-porphyrin **5** and peptide **13**, in the presence of CuSO_4_ and ascorbic acid in DMSO in fact gave the desired conjugate again in very high yield (78%). Thus, even incorporating the azido function directly at the N-terminus of the polyfunctional CPP sequence (mutating Gly to azidoacetyl) appeared to be well tolerated for this ligation.

#### Strain-promoted azide–alkyne cycloaddition (SPAAC)

While it was found to be possible to efficiently ligate simple azido or alkynyl porphyrins to the N-terminus of a complementary Tat peptide in either sense *via* CuAAC chemistry, a general drawback of this approach is the need to protect the porphyrin macrocycle *via* pre-formation of a Zn complex. Furthermore, preliminary studies with other porphyrin and chlorin derivatives suggested that significant reaction optimisation might be required on a case-by-case basis depending on the precise photosensitiser structure.^43^

Recent advances in biorthogonal ligation chemistry^44–46^ have yielded a range of novel variants on the original triazole approach in which the use of a strained alkyne component removes the need for copper catalysis, and this has indeed recently been shown to be suitable for generating specific porphyrin–antibody conjugates.^36^ We chose to investigate SPAAC ligations for the effective synthesis of both N- and C-terminally linked CPP–porphyrin conjugates, and as described above, porphyrin derivative **8** bearing a strained dibenzocyclooctyne (DBCO) function could be readily prepared by acylation of **2** with the commercially available carboxylic acid derivative. Owing to the chemical reactivity of the DBCO unit, the preferred sense of combination was with the azido function in the peptide component as detailed above, either through N-terminal acylation with a suitable azidoalkanoic acid (Tat peptide **12**), or by incorporation of a C-terminally located azidolysine (peptides **16–18**). Reaction of 2 eq. of DBCO porphyrin **8** with the N-terminal azido Tat peptide **12** proceeded smoothly in DMSO to give the expected conjugate **26** in 65% yield as an inseparable mixture of triazole regioisomers.^44^ Again there was no significant loss of efficiency upon switching from an N- to C-terminal ligation: combination of **8** with the relevant C-terminally ligatable Tat peptide **16** also proceeded effectively to give conjugate **27** in 50% yield after HPLC isolation. When **8** was combined with the penetratin and pVEC derivatives **17** and **18**, once again the desired C-terminally ligated conjugates **28** and **29** were obtained with high efficiency irrespective of the length of the peptide component and without requiring a significant excess of **8** (see Scheme 3).

### Uptake and subcellular localisation of CPP conjugates

**Fig. 2:**
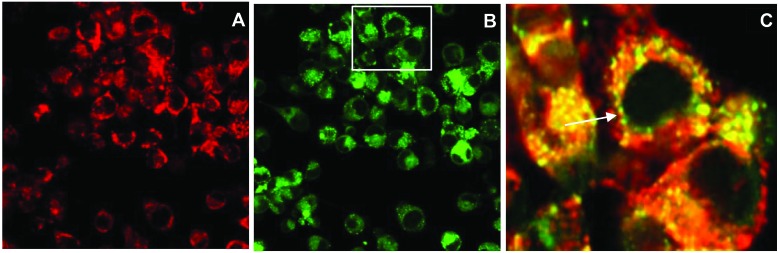
Cellular uptake and colocalisation of conjugate 20 with LysoTracker Green in MC28 cells using laser scanning confocal microscopy. Cells were incubated with 20 (2.5 μM) for 24 h. LysoTracker Green (100 nM) was applied to cells 30 min before imaging. A: **20** alone, B: LysoTracker Green, C: merged A and B inset figure highlighting the overlap of fluorescence from **20** and LysoTracker Green. Scale bar: 20 μm.

The efficient uptake and endolysosomal localisation of the amphiphilic CPP-photosensitiser conjugates was determined by microscopy in MC28 rat fibrosarcoma cells. All the N- and C-terminally linked conjugates studied showed the expected sub-cellular localisation predicted for their overall amphiphilic character (see Fig. 1) and which would render them potentially suitable photosensitisers for PCI applications. Fig. 2 shows typical images demonstrating the efficient uptake of C-linked derivative **20**. This parallels the behaviour of **20** previously observed in the clinically important HN5 head and neck cancer cell line, wherein greatly enhanced cellular uptake of the porphyrin moiety is provided by the function of the CPP to which it is conjugated.^18^ Endolysosomal localisation of the conjugate was confirmed by colocalisation of the porphyrin fluorescence with Lysotracker Green as visualised by the yellow colour in the merged image in Fig. 2C. All the conjugates were highly water soluble, unlike the parent porphyrin **1** and functionalised but non-ligated porphyrins **2–8**.

### Phototoxicity

**Fig. 3:**
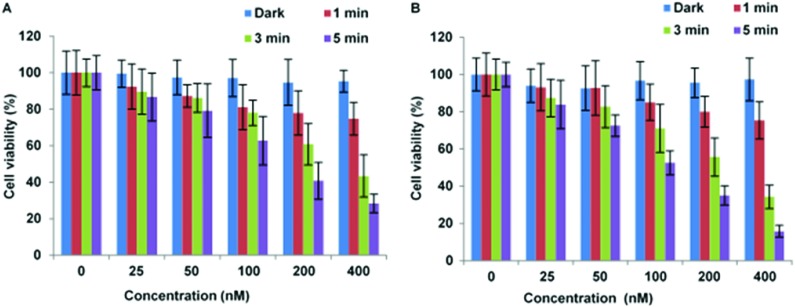
PDT effect of **19** (A) and **20** (B) in MC28 cells. Cells were incubated with Tat–porphyrin conjugates at various concentrations and were illuminated with a blue lamp for up to 5 min. MTT assay was carried out 48 h after light exposure. Data are presented as mean value ± standard deviation (SD) of three independent experiments.

The localisation of CPP-targeted porphyrin derivatives in lysosomal compartments should result in a highly efficient targeted PDT effect, if as desired, the photosensitiser is localised in the membrane of these vesicles where singlet oxygen generated can effectively photo-oxidise unsaturated lipid materials.^20^ The photoxicities of selected N- and C-linked conjugates were therefore examined in monolayer culture in MC28 and MCF-7 human breast cancer cells. A significant photo-induced reduction in cell viability was observed with both N- and C-terminally linked maleoyl conjugates **19** and **20** in MC28 cells, which was enhanced with increased photosensitiser concentration and dose of blue light. Comparison of **19** and **20** however revealed little difference in performance as a result of switching the photosensitiser location within the peptide carrier (Fig. 3).

**Fig. 4:**
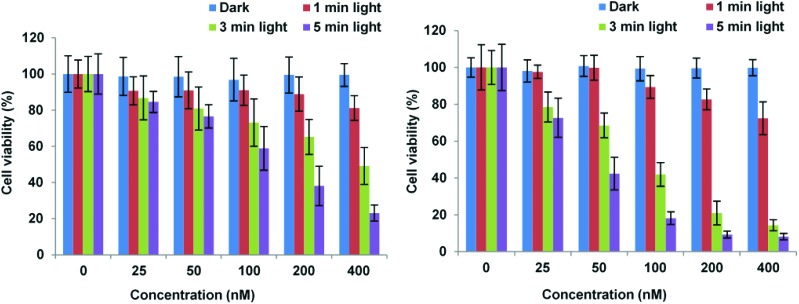
PDT effect of **24** (A) and **26** (B) in MC28 cells. Cells were incubated with Tat–porphyrin conjugates at various concentrations and were illuminated with a blue lamp for up to 5 min. MTT assay was carried out 48 h after light exposure. Data are presented as mean value ± SD of three independent experiments.

Importantly, Fig. 3 shows that control experiments in the dark for both conjugates resulted in no chemical toxicity at concentrations that were significantly greater than those typically employed for *in vitro* PCI experiments (see below). This confirmed that the observed reduction in cell viability was light-induced rather than being associated with any membrane-disrupting effects due to the CPP moiety. The triazole conjugates **24** and **26** also displayed a concentration and light dose-dependent increase in the PDT effect (Fig. 4), again without dark toxicity under the experimental conditions. Table 1 shows the porphyrin dose required to induce 50% toxicity (LD_50_) after 5 min illumination for the four conjugates **19**, **20**, **24**, and **26**. These values show that while the PDT performance is indeed similar for **19** and **20** (N- *vs*. C-terminal porphyrin conjugation), connecting the hydrophobic porphyrin and the polycationic hydrophilic peptide components *via* the more extended triazole-based linker of **26** seems to be highly beneficial. Notwithstanding this observation, the spectroscopic properties of the porphyrin unit did not appear to be affected by peptide ligation for these or any of the conjugates (see ESI†).

**Table 1:** LD_50_ values for PDT for Tat-porphyrin conjugates in MC28 cells following 5 min illumination. Estimated errors are SDs

Compound	LD_50_ PDT (nM)	LD_50_ dark (μM)
**19**	114 ± 8	≫1
**20**	94 ± 6	≫1
**24**	108 ± 6	≫1
**26**	37 ± 2	≫1

**Fig. 5:**
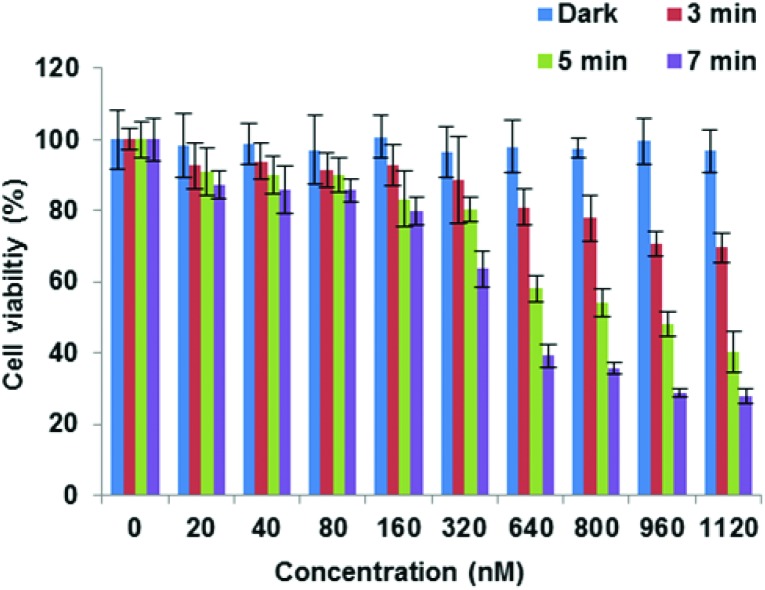
PDT effect of **29** in MCF-7 cells. Cells were incubated with photosensitiser at various concentrations and were illuminated with a blue lamp for up to 7 min. The MTT assay was carried out 48 h after light exposure. Data are presented as mean value ± SD of three independent experiments.

Finally, the C-terminally linked triazole conjugate **29** exemplifying SPAAC ligation was evaluated in MCF7 cells. Once again, an effective concentration and light dose-dependent reduction in cell viability was observed (Fig. 5), with LD_50_ = 449 nM for 7 min irradiation. As expected, no dark toxicity was shown when the cells were incubated with **29** at various concentrations up to 1 μM.

### Light-triggered drug delivery

**Fig. 6:**
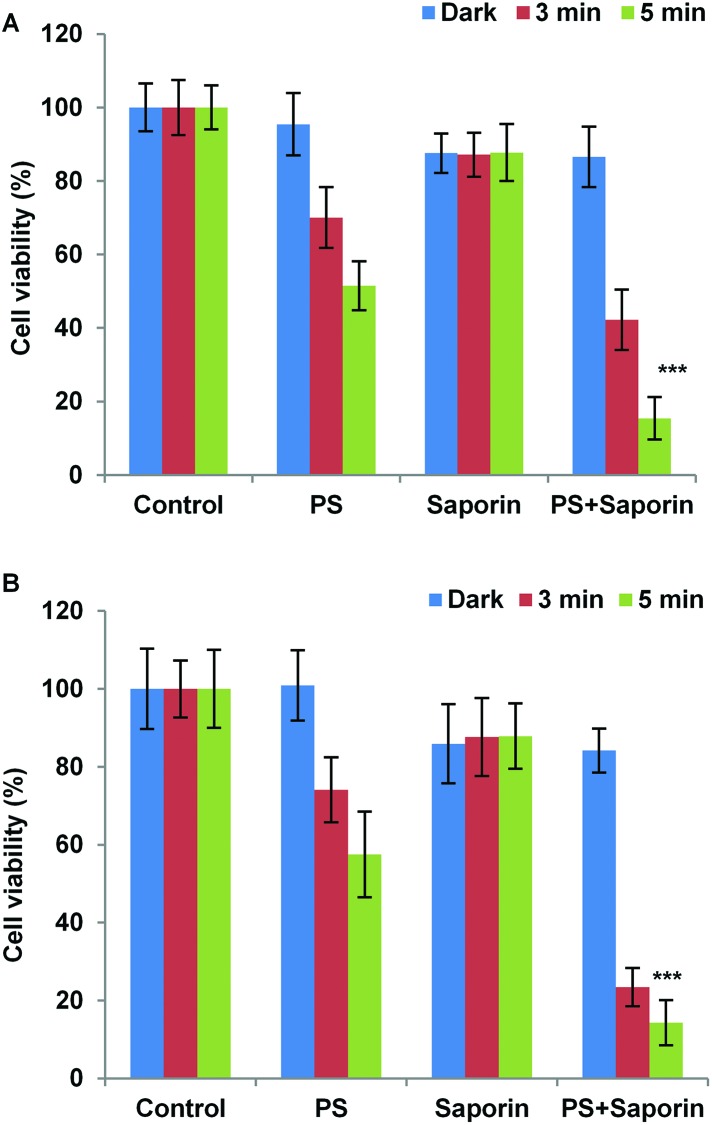
Light-induced cytotoxic response for **19** (A) and **26** (B) in MC28 cells, showing PDT (without saporin) and PCI (with saporin) effects. Cells were incubated with photosensitiser (50 nM) with or without saporin (20 nM) for 18 h. Irradiation was carried out for 3 and 5 min with a blue lamp. MTT assay was carried 72 h after irradiation. The experiments were repeated 3 times, and representative data are shown. Error bars = SD. Statistically significant difference between PCI_**conjugate19**-saporin_ *versus* PDT_**conjugate19**_ (*p* < 0.0001) is indicated by ***. Likewise, statistically significant difference between PCI_**conjugate26**-saporin_ *versus* PDT_**conjugate26**_ (*p* < 0.0001) is indicated by ***.

Two of the N-terminally linked Tat conjugates, **19** and **26** were studied further for their ability to promote light-induced relocalisation and endosomal escape of a co-administered protein toxin. Saporin is a 30 kDa ribosome inactivating protein, which has been widely used in neuronal research and as a model compound for PCI studies. Upon cellular uptake, it is ordinarily entrapped within lysosomes, thus severely limiting its toxicity. PDT treatment and PCI of saporin with **19** and **26** resulted in almost the same level of cytotoxic response for each compound (Fig. 6). This data shows that for the PCI experiments the effect of combining either conjugate with saporin is synergistic rather than additive. Indeed, with both conjugates a significant reduction in cell viability was observed in PCI-treated cells *versus* PDT-treated cells (*p* < 0.0001). For example at 5 min illumination in Fig. 6B for **26**, PCI resulted in a four-fold reduction in viability compared to PDT. PCI efficacy is defined as the ratio of the viability measured using PDT over the PCI viability, and since the reduction in viability using saporin alone is small (approximately 10% in either case) this ratio gives a measure of the enhancement in saporin cytotoxicity induced by PCI.^47^ In the absence of effective light-triggered endolysosomal disruption and saporin release, the PDT : PCI ratio would be near unity. The ratios observed here of 3.3 for **19** and 4.0 for **26** showing that effective PCI is induced and to similar extents in both conjugates, notwithstanding the difference in the CPP-photosensitiser linkage.

## Experimental

### General information

Chemical reagents were purchased from Sigma-Aldrich, Fluka, Acros, Novabiochem, and Bachem. Peptide grade DMF was purchased from Rathburn Chemicals. Anhydrous DCM was obtained by distillation over calcium hydride. Dowex-1 basic anion exchange resin 100–200 mesh (hydroxide form) was purchased from Sigma and was activated by overnight soaking in 2 M aq. NaOH; the beads were then washed with deionised water and with MeOH. All other solvents were purchased from Fisher Scientific and used as received. Analytical TLC was performed using silica gel 60 F_254_ pre-coated on aluminium sheets (Merck). Column chromatography was performed on silica gel 60 (35–70 micron) from Sigma-Aldrich. Melting points were recorded on an Electrothermal IA9200 melting point apparatus in open capillaries, and are quoted uncorrected. UV spectra were recorded on a Perkin-Elmer Lambda 19 uv/vis spectrophotometer. Fluorescence spectra were recorded on a Cary Eclipse fluorimeter. ^1^H and ^13^C NMR were recorded using a Varian Mercury-VX spectrometer at 400 MHz (^1^H) and 100 MHz (^13^C) or a Bruker Avance III 500 at 500 MHz (^1^H) and 125 MHz (^13^C). Chemical shift values are given in ppm (*δ*). *J* values are given in Hz. Analytical RP-HPLC was performed on a Dionex Ultimate 3000 system (Dionex, UK), with a VWD-3400 variable wavelength detector, and a RF-2000 fluorescence detector. Analyses were performed at 35 ± 0.1 °C on a Gemini 5 μ C18 110A column, (150 × 4.6 mm – Phenomenex, UK), equipped with a Security Guard C18 (ODS) 4 × 3.0 mm ID guard column (Phenomenex, UK), at a flow rate of 1 mL min^–1^. Mobile phase A was 0.1% aq. TFA, mobile phase B was 0.1% TFA in MeCN. (Method 1: 0.0–10.0 min 0–95% B, 10.0–15.0 min 95% B, 15.0–15.1 min at 95–5% B, 15.1–18.0 min 5% B. Method 2: 0.0–10.0 min 0–95% B, 10.0–20.0 min 95% B, 20.0–20.1 min at 95–5% B, 20.1–23.0 min 5% B). Preparative RP-HPLC was performed on a Dionex HPLC system equipped with a Phenomenex Gemini 5 μ C18 (250 × 10 mm) column at a flow rate of 2.5 mL min^–1^. Solid phase extractions were performed with Supelco Discovery DSC-18 cartridges (2 g). High resolution mass spectrometry was performed using a Bruker MicroTOF autospec ESI mass spectrometer.

### 5-(4-Aminophenyl)-10,15,20-triphenylporphyrin (**2**)

The method of Luguya *et al.* ^26^ was modified as follows. *Nitration*: a solution of *meso*-tetrakistetraphenylporphyrin **1** (1.60 g, 2.60 mmol) in TFA (80 mL) in an air-open round-bottom flask was treated at 18 °C with NaNO_2_ (321 mg, 4.64 mmol). After 3 min, the reaction was quenched with H_2_O (40 mL), then transferred to a separatory funnel and further diluted with H_2_O (80 mL) and DCM (160 mL). After separation, the aqueous phase was extracted with DCM (2 × 150 mL). The organic phases were combined, washed with saturated aq. NaHCO_3_ (2 × 175 mL) and brine (175 mL), then dried over MgSO_4_. The combined organic phases were filtered and evaporated to give a residue (1.89 g), which was used directly in the next step. *Reduction*: a solution of the preceding crude mixture of unreacted **1** and mono- and dinitrated products in DCM (640 mL) and MeOH (160 mL) in an air-open round-bottom flask was treated with 5% Pd/C (320 mg). NaBH_4_ (241 mg, 63.7 mmol) was added in small portions with stirring during 10 min, then the mixture was stirred for an additional 15 min. The reaction was quenched by the addition of H_2_O (150 mL), and the resulting suspension was transferred to a separatory funnel. The organic phase was separated, washed with brine (100 mL), dried over MgSO_4_, filtered and evaporated. The crude product was purified by column chromatography, eluting first with toluene to remove unreacted **1** (589 mg, 37% recovery), then 0–10% EtOAc in DCM to elute **2** (564 mg, 34% from **1**) and then diaminoporphyrins (368 mg, 22% from **1**). The spectroscopic data (^1^H NMR, ^13^C NMR) were indistinguishable from the literature.^26^

### Preparation of ligatable porphyrins (**3**)–(**8**)

Compounds **3** and **5–7** were prepared according to the general procedure detailed below.

*General procedure*: a stirred solution of amino derivative **2** (0.08 mmol) in anhydrous DCM (2.5 mL) was treated with the corresponding carboxylic acid (0.16 mmol), followed by EDC (0.16 mmol), HOBt (0.16 mmol) and DIPEA (0.47 mmol). The solution was protected from light and stirred under nitrogen at room temperature overnight. The reaction mixture was diluted with DCM, washed with 0.1 M HCl, saturated aq. NaHCO_3_, and brine. The organic phase was dried (MgSO_4_), filtered, and the solvent was evaporated. The crude product was purified by column chromatography (0–20% EtOAc/DCM gradient).

#### 5-[4-(3-Maleimido)-propionylamidophenyl]-10,15,20-triphenylporphyrin (**3**)^17,37^

Scale: 0.08 mmol. Dark purple solid (43 mg, 69%). mp 278–280 °C; UV-Vis (CHCl_3_), nm (%): 418 (100), 515 (6.8), 550 (5.1), 590, (4.1), 646 (3.7); ^1^H NMR (400 MHz, (CD_3_)_2_SO) *δ* 8.88 (m, 8H), 8.28–8.19 (m, 10H), 7.90 (m, 9H), 7.14 (s, 2H), 3.90 (t, *J* = 9.6, 2H), 2.77 (t, *J* = 9.6, 2H), –2.85 (s, 2H); ^13^C NMR (100 MHz, (CD_3_)_2_SO) *δ* 170.9, 169.0, 141.2, 135.9, 134.7, 134.2, 129.0, 128.6, 128.1, 127.0, 120.0, 119.9, 117.7, 42.1, 35.9, 34.0; HRMS [found (ESI+) 781.2927 [M + H]^+^, C_51_H_37_N_6_O_3_ requires 781.2916].

#### 5-{4-[2-(Bis-*tert*-butoxycarbonylaminooxy)-acetyl]-amidophenyl}-10,15,20-triphenylporphyrin (**4**)

A solution of the amino derivative **2** (0.10 mmol) in DCM (2 mL) was treated with 2-(bis-*tert*-butoxycarbonylaminooxy)acetic acid (0.30 mmol), followed by PyBOP (0.30 mmol) and DIPEA (0.60 mmol). The solution was protected from light and stirred under nitrogen at room temperature, until TLC showed consumption of **2**. The reaction mixture was diluted with DCM, washed with 10% aq. citric acid, saturated aq. NaHCO_3_, and brine. The organic phase was dried (MgSO_4_), filtered, and the solvent was evaporated. The crude product was purified by column chromatography (EtOAc/40–60 petrol gradient) and recrystallised from DCM/MeOH to give **4** as a dark purple solid (60 mg, 67%); mp 289–291 °C; UV-vis (CHCl_3_), nm (%): 418 (100), 515 (6.7), 549 (4.5), 590 (4.5), 590 (3.8), 646 (3.6); ^1^H NMR (400 MHz, CDCl_3_) *δ* 10.25 (s, 1H), 8.87–8.81 (m, 8H), 8.20–8.17 (m, 8H), 8.05 (d, *J* = 6.4, 2H), 7.76–7.68 (m, 9H), 4.71 (s, 2H), 1.61 (s, 18H), –2.76 (s, 2H); ^13^C NMR (100 MHz, CDCl_3_) *δ* 167.0, 166.3, 150.9, 142.2, 135.1, 134.5, 127.7, 126.7, 120.1, 119.6, 118.0, 85.7, 28.1; HRMS [found (ESI+) 903.3817 [M + H]^+^, C_56_H_51_N_6_O_6_ requires 903.3870].

#### 5-[4-(4-Pentynoyl)-amidophenyl]-10,15,20-triphenylporphyrin (**5**)

Scale: 0.11 mmol. Dark purple solid (42 mg, 52%); mp 277–279 °C; UV-Vis (CHCl_3_), nm (%): 418 (100), 515 (9.1), 550 (7.0), 589 (6.2), 645 (5.8); ^1^H NMR (500 MHz, (CD_3_)_2_SO) *δ* 10.45 (s, 1H), 8.89 (m, 8H), 8.24–8.07 (m, 10H), 7.89–7.83 (m, 9H), 2.92 (s, 1H), 2.74 (m, 2H), 2.62 (m, 2H), –2.91 (s, 2H); ^13^C NMR (100 MHz, (CD_3_)_2_SO) *δ* 170.9, 169.5, 142.1, 138.3, 137.4, 135.5, 128.5, 128.3, 127.7, 126.7, 120.2, 118.1, 70.0, 69.5, 62.8, 36.5; HRMS [found (ESI+) 710.2907 [M + H]^+^, C_49_H_36_N_5_O requires 710.2920].

#### 5-[4-(11-Azido)-undecanoylamidophenyl]-10,15,20-triphenylporphyrin (**6**)

Scale: 0.11 mmol. Dark purple solid (37 mg, 40%); mp 221-224 °C; UV-vis (CHCl_3_), nm (%): 419 (100), 515 (5.6), 549 (5.0), 589 (3.2), 646 (2.9); ^1^H NMR (400 MHz, (CD_3_)_2_SO) *δ* 10.36 (s, 1H), 8.97–8.82 (m, 8H), 8.27–8.11 (m, 10H), 7.92–7.83 (m, 9H), 3.32 (t, *J* = 6.8, 2H), 2.50 (t, *J* = 7.2, 2H), 1.77 (m, 2H), 1.54 (m, 2H), 1.42–1.22 (m, 12H), –2.82 (s, 2H); ^13^C NMR (100 MHz, (CD_3_)_2_SO) *δ* 171.7, 141.2, 139.4, 135.6, 134.7, 134.2, 131.2, 128.0, 127.0, 120.1, 119.9, 119.8, 117.4, 50.6, 36.6, 28.9, 28.8, 28.8, 28.7, 28.5, 28.2, 26.1; HRMS [found (ESI+) 839.4185 [M + H]^+^, C_55_H_51_N_8_O requires 839.4186].

#### 5-[4-(11-Azido)-butanoylamidophenyl]-10,15,20-triphenylporphyrin (**7**)

Scale: 0.08 mmol. Dark purple solid (37 mg, 62%); mp 200–201 °C dec.; UV-Vis (CHCl_3_), nm (%): 419, 549, 589; ^1^H NMR (400 MHz, CDCl_3_) *δ* 8.88–8.80 (m, 8H), 8.22–8.19 (m, 6H), 8.13 (d, *J* = 8.0, 2H), 7.81–7.72 (m, 9H), 7.41 (s, 1H), 3.50 (t, *J* = 6.5, 2H), 2.52 (t, *J* = 6.5, 2H), 2.11 (m, 2H), –2.75 (br, 2H); ^13^C NMR (100 MHz, CDCl_3_) *δ* 170.2, 142.0, 138.1, 137.3, 135.1, 134.5, 127.7, 126.7, 120.2, 119.4, 117.9, 60.4, 50.7, 34.1, 24.6, 21.1, 14.2; HRMS [found (ESI+) 731.3074 [M + H]^+^, C_48_H_37_N_8_O requires 731.3085].

#### 5-[4-(6-Dibenzoazacyclooctynecaproateamido)ethylamido phenyl]-10,15,20-triphenylporphyrin (**8**)

A solution of **2** (20.0 mg, 0.031 mmol) in anhydrous DCM (5 mL) was treated with HOBt (5.0 mg, 0.036 mmol), dibenzocyclooctyne-acid (10.0 mg, 0.030 mmol), EDC (7.0 mg, 0.036 mmol) and DIPEA (15.6 μL, 0.09 mmol) and was stirred at room temperature overnight. The solvent was evaporated and the crude product was purified directly by column chromatography, eluting with 10% acetone/DCM. This gave **8** as a red solid (21 mg, 70%); mp >250 °C; UV-Vis (CHCl_3_), nm: 420, 516, 553, 594, 694; ^1^H NMR (500 MHz, (CD_3_)_2_SO, 60 °C) *δ* 10.03 (s, 1H), 8.79–8.84 (m, 6H), 8.20 (d, *J* = 7.5, 6H), 8.11 (d, *J* = 8.0, 2H), 7.99 (d, *J* = 8.0, 2H), 7.79–7.83 (m, 9H), 7.25–7.68 (m, 9H), 5.00–5.08 (m, 1H), 3.56–3.62 (m, 1H), 2.23–2.28 (m, 3H), 1.89–1.95 (m, 1H)), 1.33–1.47 (m, 4H), –2.80 (s, 1H); ^13^C NMR (125 MHz, (CD_3_)_2_SO, 60 °C) *δ* 171.43, 171.04, 151.64, 148.19, 141.03, 141.01, 139.03, 137.38, 135.43, 134.26, 133.89, 132.19, 132.06, 130.87, 129.12, 128.58, 128.30, 128.05, 127.73, 127.65, 127.45, 127.33, 126.64, 126.48, 124.83, 121.35, 119.79, 119.66, 119.56, 117.87, 117.30, 54.58, 36.07, 33.76, 30.27, 24.38, 24.33. HRMS [found (ESI+) 945.4067 [M + H]^+^, C_65_H_48_N_6_O_2_ requires 945.3912].

### 5-[4-(2-Aminoxy)-acetylamidophenyl]-10,15,20-triphenylporphyrin (**9**)

A solution of **4** (20.0 mg, 0.02 mmol) in 50% TFA/DCM (2 mL) was stirred at room temperature for 30 min, then it was treated with activated Dowex-1 resin (hydroxide form) until the acid was neutralised. The resin was filtered off, and the solvent was evaporated. Filtration through a pad of silica gel (eluent: DCM) followed by recrystallization (DCM/MeOH), gave **9** as a dark purple solid (14 mg, 94%); mp 266–269 °C; UV-Vis (CHCl_3_), nm (%): 418 (100), 515 (6.6), 548 (4.9), 590 (4.0), 646 (3.6); ^1^H-NMR (400 MHz, CDCl_3_) *δ* 8.86–8.77 (m, 8H), 8.41 (s, 1H), 8.20–8.12 (m, 8H), 7.95–7.89 (m, 2H), 7.76–7.68 (m, 9H), 5.85 (s, 2H), 4.40 (s, 2H) –2.79 (s, 2H); ^13^C-NMR (100 MHz, CDCl_3_) *δ* 168.1, 165.4, 142.1, 138.4, 136.9, 135.1, 134.5, 131.1, 129.7, 127.7, 126.7, 120.2, 119.4, 118.1, 118.0, 75.3; HRMS [found (ESI+) 703.2791 [M + H]^+^, C_46_H_34_N_6_O_2_ requires 703.2821].

### Preparation of porphyrin Zn complexes

#### 5-[4-(4-Pentynoyl)-amidophenyl]-10,15,20-triphenyl porphyrinato zinc(ii) (**Zn-5**)

A solution of porphyrin **5** (42 mg, 0.056 mmol) in DCM (10 mL) was treated with Zn(OAc)_2_·2H_2_O (63.0 mg, 0.288 mmol) in MeOH (1 mL) and the mixture was stirred at 40 °C for 10 min then at room temperature until TLC analysis showed complete conversion of the starting material. The solution was then diluted with DCM (50 mL) and washed with brine. The organic phase was dried (MgSO_4_), filtered, and the solvent was evaporated to give (**Zn-5**) as a dark magenta solid (45 mg, quant.); mp >300 °C; UV-Vis (CHCl_3_), nm (%): 420 (100), 548 (7.6), 587 (4.6); ^1^H-NMR (400 MHz, (CD_3_)_2_SO) *δ* 10.56 (s, 1H), 8.93–8.80 (m, 8H), 8.27–8.12 (m, 10H), 7.87–7.83 (m, 9H), 2.97 (t, *J* = 2.4, 1H), 2.82–2.78 (m, 2H), 2.70–2.66 (m, 2H); ^13^C-NMR (100 MHz, (CD_3_)_2_SO) *δ* 170.0, 149.5, 149.3, 149.2, 142.8, 138.6, 137.5, 134.5, 134.2, 131.7, 131.5, 127.4, 126.6, 120.3, 120.2, 117.2, 83.8, 71.6, 35.5, 14.3.

#### 5-[4-(11-Azido)-undecanoylamidophenyl]-10,15,20-triphenyl porphyrinato zinc(ii) (**Zn-6**)

As for (**Zn-5**), but with **6** (18 mg, 0.02 mmol). **Zn-6** was obtained as a dark purple solid (19 mg, 97%); mp 169–172 °C; UV-Vis (CHCl_3_), nm (%): 420 (100), 548 (7.8), 585 (4.7); ^1^H-NMR (500 MHz, (CD_3_)_2_SO) *δ* 10.32 (s, 1H), 8.88–8.80 (m, 8H), 8.26–8.05 (m, 8H), 7.85 (m, 9H), 7.62 (m, 2H), 1.76 (m, 2H), 1.59 (m, 2H), 1.45–1.35 (m, 16H); ^13^C-NMR (125 MHz, (CD_3_)_2_SO) *δ* 176.5, 171.6, 149.5, 149.2, 149.2, 142.7, 138.8, 137.2, 134.5, 134.1, 132.0, 131.7, 131.5, 131.4, 128.8, 128.7, 127.4, 126.6, 120.2, 120.2, 117.1, 50.6, 36.6, 28.9, 28.9, 28.8, 26.0, 25.1, 22.4, 22.3.

#### 5-[4-(11-Azido)-butanoylamidophenyl]-10,15,20-triphenylporphyrin zinc(ii) (**Zn-7**)

As for (**Zn-5**), but with **7** (18.5 mg, 0.025 mmol). (**Zn-7**) was obtained as a dark purple solid (15 mg, 71%); mp 181–183 °C; UV-Vis (CHCl_3_), nm (%): 420 (100), 547 (7.5), 584 (4.4); ^1^H NMR (400 MHz, (CD_3_)_2_SO) *δ* 10.41 (s, 1H), 8.87–8.82 (m, 8H), 8.25–8.07 (m, 10H), 7.87–7.83 (m, 9H), 3.58 (t, *J* = 6.8, 2H), 2.65 (m, 4H), 2.05 (q, *J* = 7.0, 2H); ^13^C-NMR (125 MHz, (CD_3_)_2_SO) *δ* 170.7, 149.5, 149.3, 149.2, 142.8, 138.7, 137.3, 134.5, 134.2, 131.7, 131.6, 127.5, 126.6, 120.3, 120.2, 117.2, 50.4, 33.5, 24.4.

### Peptide synthesis

All peptides were assembled according to the Fmoc strategy, on Rink Amide MBHA Resin (Novabiochem, 200–400 mesh, 0.60 mmol g^–1^ loading) using an Activo P11 automated synthesiser fitted with a reactor heating jacket. The synthesis was performed on 250 mg of resin (0.15 mmol scale). Removal of the Fmoc group from the resin was performed with 20% piperidine/DMF (2.5 mL, 4 × 3 min at 60 °C). The first residue attachment for peptides starting with a Gly, Arg, or azidolysinyl residue was performed manually at room temperature using 5 min preactivation then 1 h coupling with the amino acid (4 eq.), DIC (4 eq.) and DIPEA (6 eq.). This was followed by an acetylation step (Ac_2_O/DIPEA/DMF = 1/1/8, 2.5 mL, 1 × 10 min). For peptide **15** with C-terminal Cys, a low-racemisation protocol by Angell *et al.* was employed,^48^ utilising a 1 h coupling at room temperature without preactivation with Fmoc-Cys(Trt)-OH (4 eq.), HATU (4 eq.), HOBt (4 eq.) and collidine (4 eq.). Subsequent Fmoc deprotection steps were performed using 25% piperidine/DMF (3 mL, 1 × 5 min, 1 × 10 min), with the chain elongation steps being performed at 60 °C for 35 min using 3 eq. of each Fmoc-protected amino acid (Fmoc-Arg(Pmc)-OH, Fmoc-Gln(Trt)-OH), Fmoc-Lys(Boc)-OH, or Fmoc-Gly-OH), PyBOP (3 eq.), and DIPEA (6 eq.) in DMF (4.3 mL) (35 min,). The Fmoc deprotection step was omitted after the coupling of the final residue. The peptide resin was washed thoroughly with DMF, DCM, MeOH and Et_2_O, and dried *in vacuo*.

#### General procedure for the preparation of N-terminally ligatable peptides (**10**)–(**12**), and (**14**) from resin-bound Tat(48–57) peptide

These transformations were carried out manually, using a disposable plastic reactor (Grace UK). 100 mg of peptide resin (0.019 mmol, based on the initial loading) was swollen in DCM, then the N-terminal Fmoc protection was removed as described above. The resin-bound peptide was then acylated as follows:

(**10**)–(**12**): derivatisation was carried with the appropriate acid (3 eq.), HATU (2.9 eq.), and DIPEA (6 eq.) in DMF (2 mL) for 1 h. Completion of the acylation was confirmed by the Kaiser test.

(**14**): derivatisation was carried out using pyruvic acid succinimido ester^49^ (2 eq.) and DIPEA (2 eq.) in DMF (2 mL) overnight. Completion of the acylation was confirmed as previously.

Following the acylation step, the resin beads were filtered off under vacuum, and washed thoroughly with DMF, DCM, MeOH, and Et_2_O, and dried *in vacuo*. For the cleavage and deprotection of the ligatable peptides, the acylated peptidyl resin was swollen in DCM for 20 min, then it was treated with TFA/TIS/H_2_O (95/2.5/2.5) for 4 h. Cysteine-containing peptide (**10**) was instead treated with TFA/TIS/H_2_O/EDT (95/2.5/2.5/1) for 3 h. The resin beads were filtered off, washed with TFA, and the combined filtrates were evaporated to a small volume then anhydrous Et_2_O was added. The resulting precipitate was collected by centrifugation and was washed twice more with Et_2_O. The precipitated material was dissolved in 1% aq. TFA, filtered using a 0.2 μm syringe filter and the resulting solution was directly purified by semi-preparative HPLC. The purified peptides were then freeze-dried.

(**10**) (24 mg, 40%); HPLC: *t*_R_ (Method 1): 4.07 min; HRMS [found (ESI+) 749.9608 [M + 2H]^2+^, C_58_H_117_N_33_O_12_S_2_ requires 749.9635].

(**11**) (26 mg, 58%); HPLC: *t*_R_ (Method 1): 3.76 min; HRMS [found (ESI+) 738.4662 [M + 2H]^2+^, C_60_H_116_N_32_O_12_ requires 738.4720].

(**12**) (24 mg, 55%); HPLC: *t*_R_ (Method 1): 6.53 min; HRMS [found (ESI+) 803.0359 [M + 2H]^2+^, C_66_H_131_N_35_O_12_ requires 803.0353].

(**14**) (16 mg, 35%); HPLC: *t*_R_ (Method 1): 3.91 min; HRMS [found (ESI+) 489.3067 [M + 3H]^3+^, C_58_H_115_N_32_O_13_ requires 489.3102].

#### Preparation of ligatable peptide (**13**) *via* on-resin diazo transfer

100 mg of peptide resin was swollen in DCM and the N-terminal Fmoc deprotection was removed as described above. The resin was washed, and then swollen again in mixture of DCM/MeOH/H_2_O (2/2/1) for 1 h. The resin was then treated with imidazole-1-sulfonyl azide hydrochloride (121 mg, 0.7 mmol) DIPEA (273 μL, 1.40 mmol), and CuSO_4_·5H_2_O (17 mg, 0.07 mmol) in DCM/MeOH/H_2_O (2/2/1, 2 mL). The resin was shaken overnight, then the solution was discarded and the beads were washed with deionised water, MeOH, and DCM. Cleavage from the resin and isolation was carried out as described above to give **12** (24 mg, 55%), *t*_R_ (Method 1): 3.85 min; HRMS [found (ESI+) 474.6345 [M + 3H]^3+^, C_55_H_111_N_34_O_11_ requires 474.6385].

#### Preparation of C-terminally ligatable peptides (**15**)–(**18**)

For peptides **16–18**, the azidolysine-containing Tat, penetratin and pVec sequences were assembled as described above on Rink Amide MBHA resin (0.25 g, 0.60 mmol g^–1^ loading). For each ligatable peptide, a sample of the peptide resin (50 mg) was swollen in DCM, then the N-terminal Fmoc protection was removed. Cleavage from the resin and side chain deprotection were achieved by treatment with TFA/TIS/H_2_O (95/2.5/2.5/1) (3 mL). All the solvents were evaporated under vacuum and the residue was dissolved in TFA and cold Et_2_O was added. The white solid precipitated was isolated by centrifugation. **16** and **18** were obtained directly in >90% purity as judged by HPLC (see ESI†). For **17**, the precipitate collected by centrifugation was dissolved in 0.1% aq. TFA, filtered using a 0.2 μm syringe filter and the resulting solution was directly purified by semi-preparative HPLC. The purified peptide were then freeze-dried. C-terminal Cys-containing peptide **15** was obtained in analogous fashion to the N-terminal Cys-containing peptide **10** (see above).

(**15**) (20 mg, 33%); HPLC: *t*_R_ (Method 1): 3.90 min; compound was previously described.^9^

(**16**) (54 mg, 67%). HPLC *t*_R_ (Method 1) 7.61 min; [Found (ESI+) 609.7098 [M + 3H]^3+^, C_74_H_138_N_39_O_16_ requires 609.7056].

(**17**) (33 mg, 65%). HPLC *t*_R_ (Method 1) 5.06 min; [Found (ES+) 557.8337 [M + 2H]^2+^, C_48_H_85_N_21_O_8_S requires 557.8330].

(**18**) (40 mg, 59%). HPLC *t*_R_ (Method 1) 5.43 min; [Found (ESI+) 745.8039 [M + 3H]^3+^, C_98_H_179_N_40_O_20_ requires 745.8076].

### Ligation chemistry

#### N-terminal thiol-maleimide ligation-preparation of (**19**)

A solution of peptide Cys-Tat peptide **9** (9.1 mg, 3.6 μmol) and maleoyl-porphyrin **3** (6.0 mg, 7.7 μmol) in DMSO (1.5 mL) was treated with pyridine (73 μL) and stirred at room temperature overnight, shielded from light. The mixture was diluted with 1.0% aq. TFA and directly purified by semi-preparative HPLC. The purified conjugate was freeze-dried to give **19** as a dark green solid (10.8 mg, 91%). HPLC: *t*_R_ (Method 2): 7.12 min; UV-Vis (0.1% aq. TFA), nm (%): 438 (100), 657 (14.6); Fluorescence *λ*_max._ (0.1% aq. TFA, *λ*_exc._ = 420 nm) 687 nm; HRMS [found (ESI+) 760.7358 [M + 3H]^3+^, C_109_H_154_N_39_O_15_S requires 760.7406].

#### C-terminal thiol-maleimide ligation-preparation of (**20**)

A solution of Tat-GYKC peptide **15** (39.1 mg, 13.1 μmol) and maleoyl-porphyrin **3** (25.0 mg, 32.0 μmol) in DMSO (1.5 mL) was treated with pyridine (150 μL) and stirred at room temperature overnight, shielded from light. The mixture was diluted with Et_2_O and TFA (350 μL) was added. A mixture of conjugated and unconjugated peptide was precipitated, which was collected by centrifugation and dissolved in 1.0% aq. TFA (10 mL). This solution was then directly purified by semi-preparative. The purified conjugate was freeze-dried to give **20** as a dark green solid (37.0 mg, 75%). HPLC: *t*_R_ (Method 2): 6.95 min; UV-Vis (0.1% aq. TFA), nm (%): 423 (100), 518 (13.5); Fluorescence *λ*_max._ (0.1% aq. TFA, *λ*_exc._ = 420 nm) nm (%): 651 (100), 714 (26); HRMS [found (ESI+) 876.4583 [M + 3H]^3+^, C_126_H_178_N_43_O_19_S requires 876.4663].

#### Oxime ligation – preparation of (**21**)

A solution of pyruvoyl-Tat peptide **14** (3 mg, 1.3 μmol) and aminoxy-porphyrin **9** (4 mg, 5.7 μmol) in DMSO (500 μL) was treated with TFA (5 μL), and the mixture was allowed to stand at room temperature overnight, shielded from light. The mixture was applied to a solid phase extraction cartridge, which was washed with 0.1% aq. TFA, then 0.1% TFA in H_2_O/MeCN (80/20) and then the desired conjugate was eluted with 0.1% TFA in H_2_O/MeCN (50/50). The eluate obtained was evaporated to a small volume and the desired conjugate was isolated by semi-preparative HPLC and freeze-drying, to give **20** (3.3 mg, 75%). HPLC: *t*_R_ (Method 2): 6.91 min; UV-Vis (0.1% aq. TFA), nm (%): 439 (100), 658 (17.2); Fluorescence *λ*_max._ (0.1% aq. TFA, *λ*_exc._ = 420 nm), 684 nm; HRMS [found (ESI+) 1076.0920 [M + 2H]^2+^, C_104_H_146_N_38_O_14_ requires 1076.0948].

#### N-terminal CuAAC ligations – preparations of (**22**)–(**25**)

(**22**): a solution of azidoundecanoyl-Tat peptide (**12**) (2.0 mg, 0.79 μmol) in DMSO (400 μL), *t*BuOH (60 μL) and H_2_O (50 μL) was treated with alkynyl-TPP (**Zn-5**) (2.0 mg, 2.6 μmol), CuSO_4_·5H_2_O (12.4 μL of a 1 M solution in H_2_O, 12.4 μmol) and sodium ascorbate (20 mg, 101 μmol). The mixture was allowed to stand at room temperature, shielded from light for 1.5 h, then it was diluted with 5.0% aq. TFA, left to stand at room temperature for 30 min and directly purified by semi-preparative HPLC. The purified conjugate was freeze-dried, to give **22** as a dark green solid (1.2 mg, 45%). HPLC: *t*_R_ (Method 2): 7.67 min; UV-Vis (0.1% aq. TFA), nm (%): 439 (100), 657 (17.2); Fluorescence *λ*_max._ (0.1% aqueous TFA, *λ*_exc._ = 420 nm) 689 nm; HRMS [found (ESI+) 771.7775 [M + 3H]^3+^, C_115_H_163_N_40_O_13_ requires 771.7847].

(**23**): a solution of azidopeptide 13 (4.0 mg, 1.7 μmol) in DMSO (500 μL) was treated with alkynyl-porphyrin (**Zn-4**) (6.0 mg, 7.7 μmol), CuSO_4_·5H_2_O (1 mg, 8 μmol) and ascorbic acid (2.5 mg, 14 μmol). The mixture was allowed to stand at room temperature, shielded from light for 1.5 h, then it was applied to a solid phase extraction cartridge. The cartridge was washed with 0.1% aq. TFA, then 0.1% TFA in H_2_O/MeCN (80/20), and then the desired conjugate was eluted with 0.1% TFA in H_2_O/MeCN, (50/50). The eluate obtained was evaporated to dryness, then the residue was dissolved in 20% aq. TFA and the resulting solution allowed to stand at room temperature until HPLC analysis indicated complete decomplexation of the porphyrin moiety. The solution was then evaporated to a small volume and the desired conjugate was isolated by semi-preparative HPLC and freeze-drying, to give 16 as a dark green solid (4.1 mg, 79%). HPLC: *t*_R_ (Method 2): 6.83 min; UV-Vis (0.1% aq. TFA), nm (%): 438 (100), 657 (15.4); Fluorescence *λ*_max._ (0.1% aq. TFA, *λ*_exc._ = 420 nm) 685 nm; HRMS [found (ESI+) 1066.5903 [M + 2H]^2+^. C_104_H_145_N_39_O_12_ requires 1066.5975].

(**24**): A solution of alkynyl-Tat peptide (**11**) (1.0 mg, 0.42 μmol) in DMSO (200 μL), *t*BuOH (30 μL) and H_2_O (30 μL) was treated with azidoundecanoyl-porphyrin (**Zn-6**) (1.0 mg, 2.6 μmol), CuSO_4_·5H_2_O (6.2 μL of a 1 M solution in H_2_O, 6.2 μmol) and sodium ascorbate (10 mg, 50 μmol). The mixture was allowed to stand at room temperature, shielded from light for 3 h, then was diluted with 5.0% aq. TFA, left to stand at room temperature for 30 min and directly purified by semi-preparative HPLC. The purified conjugate was freeze-dried, to give **24** as a dark green solid (3.1 mg, 77%). HPLC: *t*_R_ (Method 2): 7.61 min; UV-Vis (0.1% aq. TFA), nm (%): 437 (100), 651 (15.2); Fluorescence *λ*_max._ (0.1% aq. TFA, *λ*_exc._ = 420 nm), 684 nm; HRMS [found (ESI+) 772.4495 [M + 3H]^3+^, C_115_H_167_N_40_O_132_ requires 772.4549].

(**25**): A solution of alkynyl-Tat peptide **11** (6.0 mg, 8.1 μmol) in DMSO (1.2 mL), *t*BuOH (180 μL) and H_2_O (180 μL) was treated with azidobutanoyl-porphyrin (**Zn-7**) (6.0 mg, 2.6 μmol), CuSO_4_·5H_2_O (37.4 μL of a 1 M solution in H_2_O, 37.4 μmol) and sodium ascorbate (60 mg, 300 μmol). The mixture was allowed to stand at room temperature, shielded from light for 3 h, then was diluted with 5.0% aq. TFA, left to stand at room temperature for 30 min and directly purified by semi-preparative HPLC. The purified conjugate was freeze-dried, to give **25** as a dark green solid (6.1 mg, 78%). HPLC: *t*_R_ (Method 2): 7.12 min; UV-Vis (0.1% aq. TFA), nm (%): 438 (100), 648 (15.9); Fluorescence *λ*_max._ (0.1% aq. TFA, *λ*_exc._ = 420 nm), 684 nm; HRMS [found (ESI+) 554.8321 [M + 4H]^4+^, C_108_H_150_N_40_O_13_ requires 554.8149].

#### N-terminal SPAAC ligation – preparation of (**26**)

A solution of azidoundecanoyl-Tat peptide **12** (7.8 mg, 4.9 μmol) and DBCO-porphyrin **8** (8.6 mg, 9.1 μmol) in DMSO (4.0 mL) was treated with pyridine (200 μL) and stirred at room temperature overnight, shielded from light. The mixture was diluted with 1.0% aq. TFA and directly purified by semi-preparative HPLC. The purified conjugate was freeze-dried to give **26** as a dark green solid (9.8 mg, 65%). HPLC: *t*_R_ (Method 2): 7.97 min; UV-Vis (0.1% aq. TFA), nm (%): 438 (100), 661 (19), 606 (3); Fluorescence *λ*_max._ (0.1% aq. TFA, *λ*_exc._ = 420 nm), 691 nm; HRMS [found (ESI+) 850.4899 [M + 3H]^3+^, C_131_H_180_N_41_O_14_ requires 850.4872].

#### C-terminal SPAAC ligations – preparation of (**27–29**)

(**27**): a solution of azidopeptide **16** (7.5 mg, 2.6 μmol) and DBCO-porphyrin **8** (5.0 mg, 5.2 μmol) in DMSO (3 mL) was stirred at room temperature overnight, shielded from light. Purification by semi-preparative HPLC gave **27** as a dark green solid (6.9 mg, 50%). HPLC: *t*_R_ (Method 2): 7.44 min; UV-Vis (0.1% aq. TFA), nm (%): 435 (100), 656 (29); Fluorescence *λ*_max._ (0.1% aq. TFA, *λ*_exc._ = 420 nm), 687 nm; HRMS [found (ESI+) 924.5006 [M + 3H]^3+^, C_139_H_186_N_45_O_18_ requires 924.5002].

(**28**): As for **27**, but with azidopeptide **17** (4.8 mg, 2.6 μmol) and DBCO-porphyrin **8** (2.6 mg, 2.8 μmol). Purification by semi-preparative HPLC gave **28** as a dark green solid (6.9 mg, 74%). HPLC: *t*_R_ (Method 2): 7.90 min; UV-vis (0.1% aq. TFA), nm (%): 433 (100), 652 (10); Fluorescence *λ*_max._ (0.1% aq. TFA, *λ*_exc._ = 420 nm), 684 nm; HRMS [found (ESI+) 697.6903 [M + 3H]^3+^, C_115_H_132_N_30_O_8_S requires 697.6850].

(**29**): As for **27**, but with azidopeptide **18** (10.0 mg, 3.3 μmol) and DBCO-porphyrin **8** (3.1 mg, 3.3 μmol) in DMSO (500 μL). Purification by semi-preparative HPLC gave **29** as a dark green solid (9.0 mg, 69%). HPLC: *t*_R_ (Method 1): 7.61 min; UV-Vis (MeOH), nm (%): 415 (100), 513 (12.96), 547 (9.50), 590 (8.23), 646 (7.81); Fluorescence *λ*_max._ (MeOH, *λ*_exc._ = 420 nm) nm (%) 653 (100), 715 (34); HRMS [found (ESI+) 795.4614 [M + 4H]^4+^, C_163_H_228_N_46_O_22_ requires 795.4529].

### Cell lines and cultivation

MC28 cells, a methylcholanthrene-induced fibrosarcoma cell line, were grown in DMEM supplemented with 10% FCS. Human breast cancer cells (MCF-7) were grown in DMEM-F12 medium containing 10% FCS. Cells were incubated at 37 °C in a humidified atmosphere containing 5% CO_2_. Unless otherwise stated materials for the cell studies were purchased from Sigma-Aldrich Gillingham, UK.

#### Cellular uptake and confocal microscopy

MC28 cells were seeded in small Petri dishes with a glass cover slip bottom (Fluorodish, World Precision Inst. UK) and allowed to attach overnight. Cells were then incubated for 24 h with conjugate **20** (2.5 μM). Afterwards, culture medium was removed and replaced with fresh medium containing LysoTracker Green (100 nM) 30 min before microscope imaging. Cells were then washed 2 times with PBS and incubated with drug-free/phenol red-free medium for the confocal imaging using an Olympus laser scanning confocal microscope (FluoView FV1000, 60× magnification, NA 1.20, Olympus UK Ltd, Essex, UK). Fluorescence from the photosensitiser was recorded within the range of 620–720 nm using a 405 nm laser for the excitation. For the LysoTracker Green imaging, cells were illuminated at 488 nm and the fluorescence signal was recorded at 500–550 nm. Colocalisation analysis and image processing were performed with ImageJ software.

#### *In vitro* PDT phototoxicity study

MC28 and MCF-7 cells were seeded out at a density of 5000 cells in 96 well plates. The cells were allowed to attach to the bottom of the wells for 24 h. The next day, cells were incubated with various concentrations of photosensitiser for 18 h. The cells were then washed twice with PBS and incubated with drug-free culture medium for 4 h. Afterwards, the cells were exposed to increasing doses of light using a blue LumiSource® flatbed lamp with peak emission at 420 nm and 7 mW cm^–2^ output (PCI Biotech, Oslo, Norway). Cell viability was evaluated 48 h after light illumination using a standard MTT (3-(4,5-dimethylthiazol-2-yl)-2,5-diphenyltetrazolium bromide) assay. Each experiment was carried out in triplicate.

#### *In vitro* PDT/PCI phototoxicity study

MC28 cells were seeded out at a density of 3000 cells in 96 well plates overnight. Cells were then treated with **19** or **26** (50 nM) or saporin (Sigma-Aldrich Gillingham, UK) (20 nM) for 18 h separately. Another group of cells were co-incubated with saporin (20 nM) and **19** or **26**. Cells were then washed twice with PBS and incubated for a further 4 h with fresh full medium. Irradiation was carried out for up to 5 min using a blue LumiSource® flatbed lamp with peak emission at 420 nm and 7 mW cm^–2^ output (PCI Biotech, Oslo, Norway). Cell viability was determined by means of the MTT assay 72 h after the light exposure. Each experiment was carried out in triplicate.

### Statistical analysis

Data were analysed using two-tailed Students *T*-test with appropriate testing *post hoc* using Prism 6 software. The dose required to reduce viability by 50% (LD_50_) was also calculated using Graphpad Prism. Error bars from the mean show ±standard deviation (SD). Values of *P* < 0.05 were considered to be significant.

## Conclusions

Bioconjugation strategies offer a highly efficient and flexible means to generate conjugates between hydrophobic porphyrins and polyfunctional CPPs under very mild conditions. These amphiphilic photosensitisers with regiospecific attachment of a tetrapyrrole component within the peptide backbone are effective agents for both targeted PDT and PCI. As exemplified in this study, strain-promoted azide–alkyne ligations offer an ideal way to repurpose simple porphyrin derivatives for PCI by attachment to a variety of CPPs. This approach opens the way for adapting clinically relevant photosensitisers with the most attractive spectroscopic properties that are currently used in PDT for minimally invasive therapies in conjunction with selected targeted CPPs.
