# Supplementary material for: Flexible synthesis of cationic peptide–porphyrin derivatives for light-triggered drug delivery and photodynamic therapy
Source: Org Biomol Chem. 2016 Nov 14;14(48):11488–501. doi: 10.1039/c6ob02135b (PMC5166568; doi:10.1039/c6ob02135b)
Supplement: Supplementary file 1 [file OB-014-C6OB02135B-s001.pdf]

Electronic Supplementary Material (ESI) for Organic & Biomolecular Chemistry.
This journal is © The Royal Society of Chemistry 2016

Electronic Supplementary Material (ESI) for Organic & Biomolecular Chemistry.
This journal is © The Royal Society of Chemistry 2016

Flexible synthesis of cationic peptide-porphyrin derivatives for light-triggered drug delivery and photodynamic therapy

R. Dondi,[a] E. Yaghini,[b] K. Tewari,[a] L. Wang,[a,c] F. Giuntini,[a] M. Loizidou,[b] A.J. MacRobert,[b] and I. M. Eggleston[a]*

[a]Department of Pharmacy and Pharmacology, University of Bath, Bath BA2 7AY, UK. E-mail: ie203@bath.ac.uk

[b]UCL Division of Surgery and Interventional Science, University College London, Royal Free Campus, Rowland Hill Street, London NW3 2PF, UK.

[c]School of Pharmaceutical Sciences, Shandong University, Jinan, Shandong 250012, China

Contents

S2: Figure 1: ^{1}H NMR, ^{13}C NMR spectra, UV-visible spectrum of **(4)**

S3: Figure 2: ^{1}H NMR, ^{13}C NMR spectra, UV-visible spectrum of **(Zn-5)**

S4: Figure 3 ^{1}H NMR, ^{13}C NMR spectra, UV-visible spectrum of **(Zn-6)**

S5: Figure 4: ^{1}H NMR, ^{13}C NMR spectra, UV-visible spectrum of **(Zn-7)**

S6: Figure 5: ^{1}H NMR, ^{13}C NMR spectra, UV-visible spectrum of **(8)**

S7: Figure 6: ^{1}H NMR, ^{13}C NMR spectra, UV-visible spectrum of **(9)**

S8: Figure 7: HPLC profile, UV-visible spectrum of **(19)**

S8: Figure 8: HPLC profile, UV-visible spectrum of **(20)**

S9: Figure 9: HPLC profile, UV-visible spectrum of **(21)**

S9: Figure 10: HPLC profile, UV-visible spectrum of **(22)**

S10: Figure 11: HPLC profile, UV-visible spectrum of **(23)**

S11: Figure 12: HPLC profile, UV-visible spectrum of **(24)**

S11: Figure 13: HPLC profile, UV-visible spectrum of **(25)**

S12: Figure 14: HPLC profile, UV-visible spectrum of **(26)**

S12: Figure 15: HPLC profile, UV-visible spectrum of **(27)**

S13: Figure 16: HPLC profile, UV-visible spectrum of **(28)**

S13: Figure 17: HPLC profile, UV-visible spectrum of **(29)**

Electronic Supplementary Material (ESI) for Organic & Biomolecular Chemistry.
This journal is © The Royal Society of Chemistry 2016

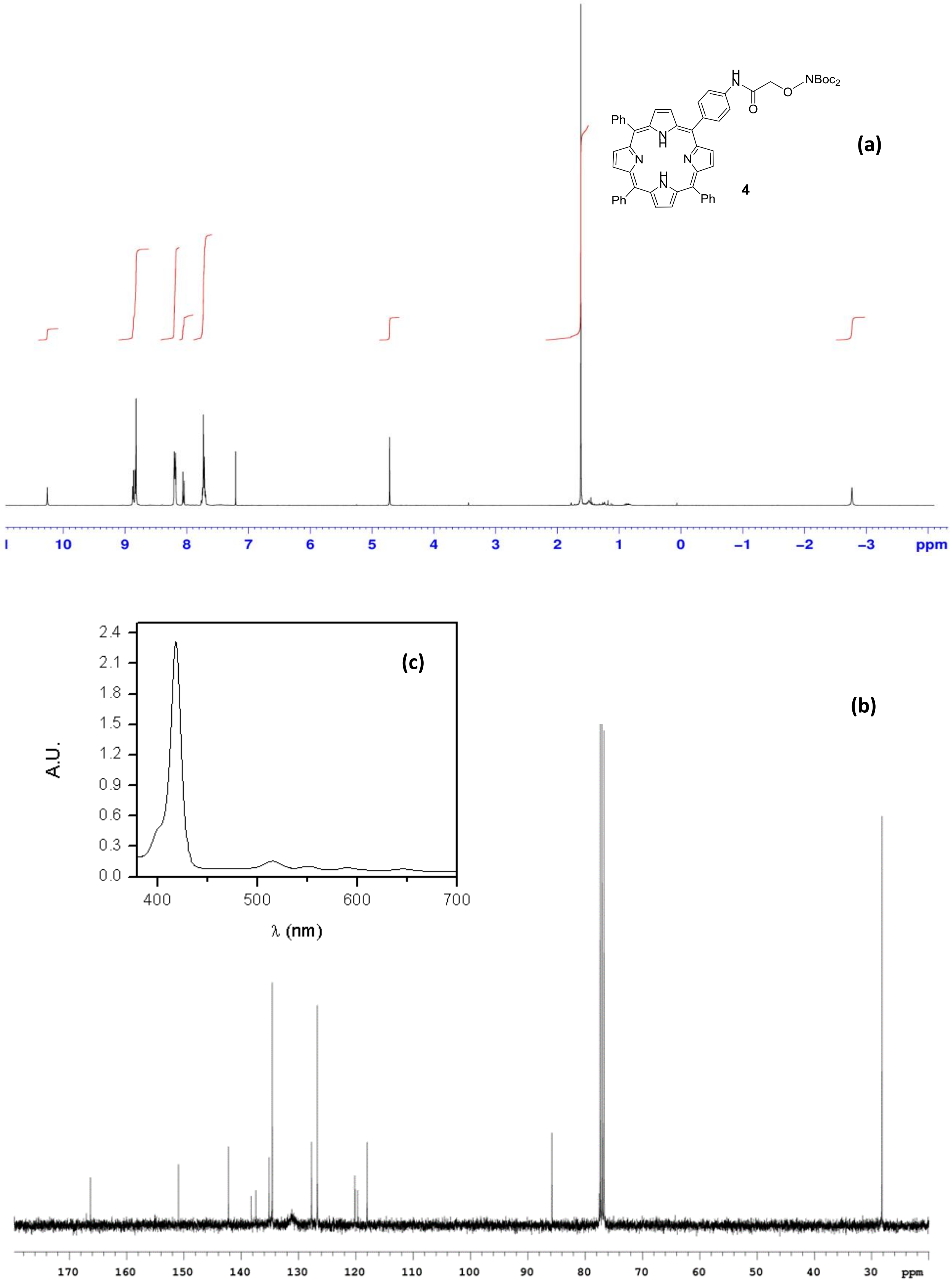

Figure 1 (a)-(b). ^{1}H NMR and ^{13}C NMR spectra in $CDCl_3$ of **4**; **(c)** UV-visible spectrum in $CHCl_3$ of **4**.

This journal is © The Royal Society of Chemistry 2016

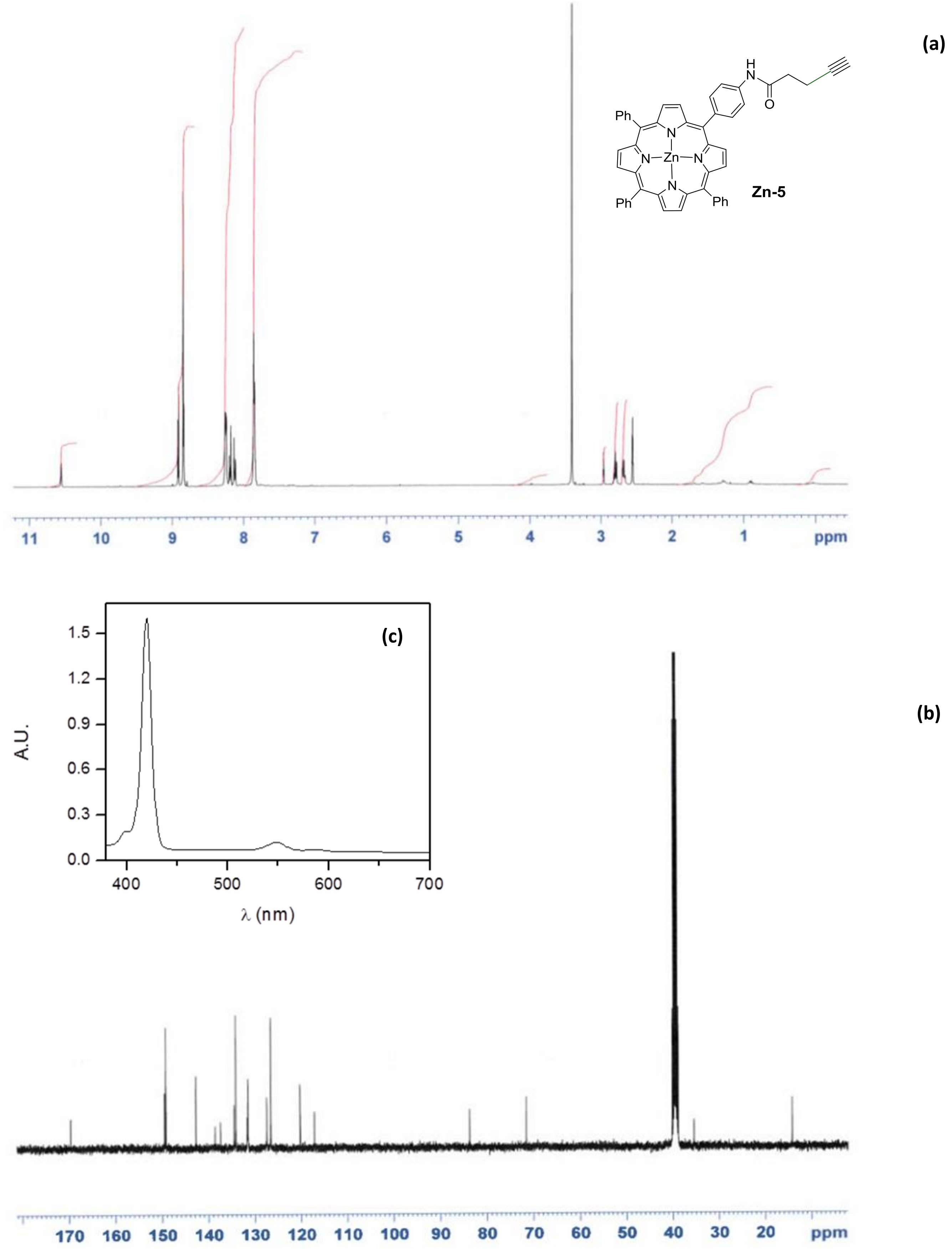

Figure 2 (a)-(b). ^{1}H NMR and ^{13}C NMR spectra in $(CD_3)_2SO$ of **Zn-5**; **(c)** UV-visible spectrum in $CHCl_3$ of **Zn-5**.

This journal is © The Royal Society of Chemistry 2016

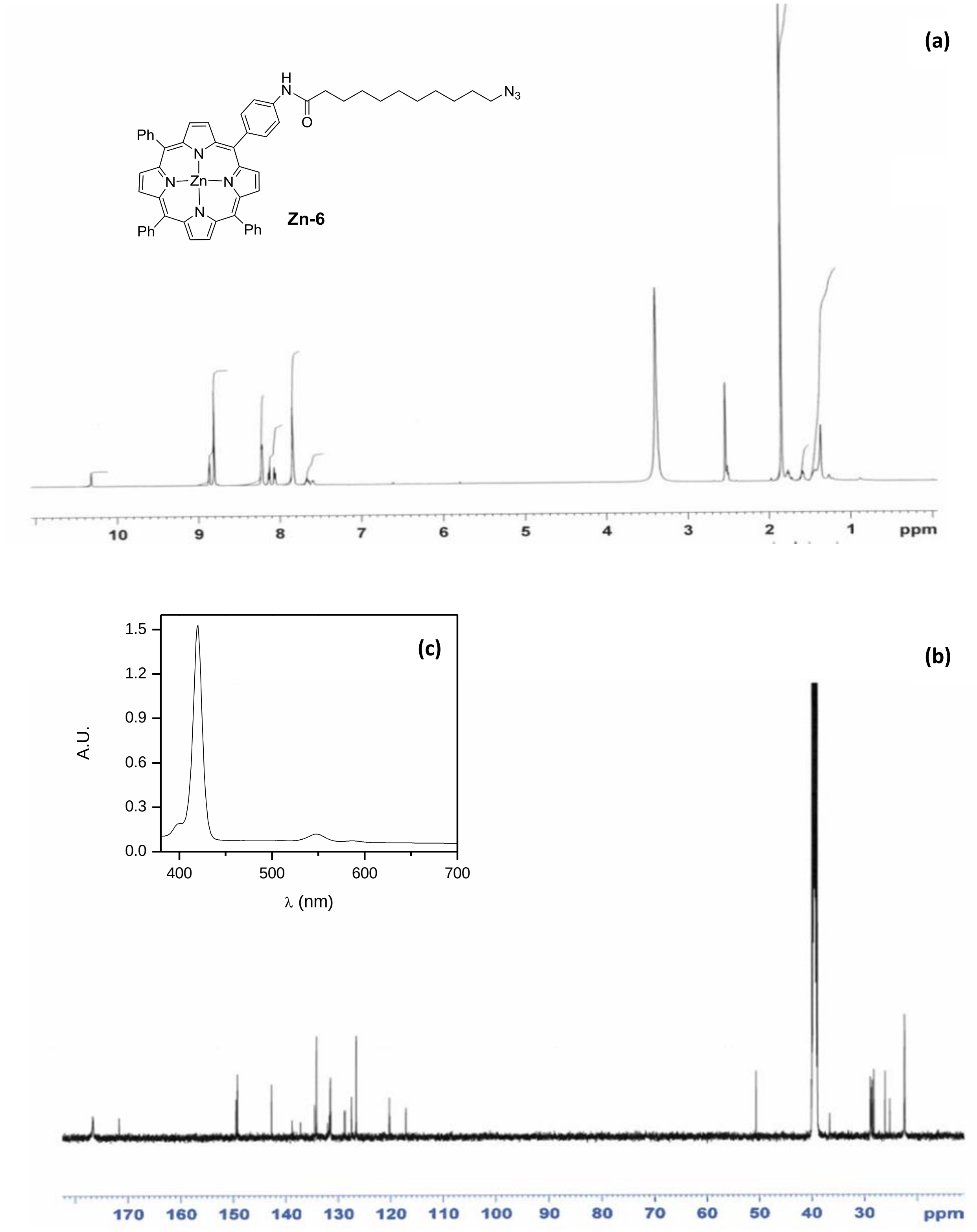

Figure 3 (a)-(b). ^{1}H NMR and ^{13}C NMR spectra in $(CD_3)_2SO$ of **Zn-6**; **(c)** UV-visible spectrum in $CHCl_3$ of **Zn-6**.

Electronic Supplementary Material (ESI) for Organic & Biomolecular Chemistry.
This journal is © The Royal Society of Chemistry 2016

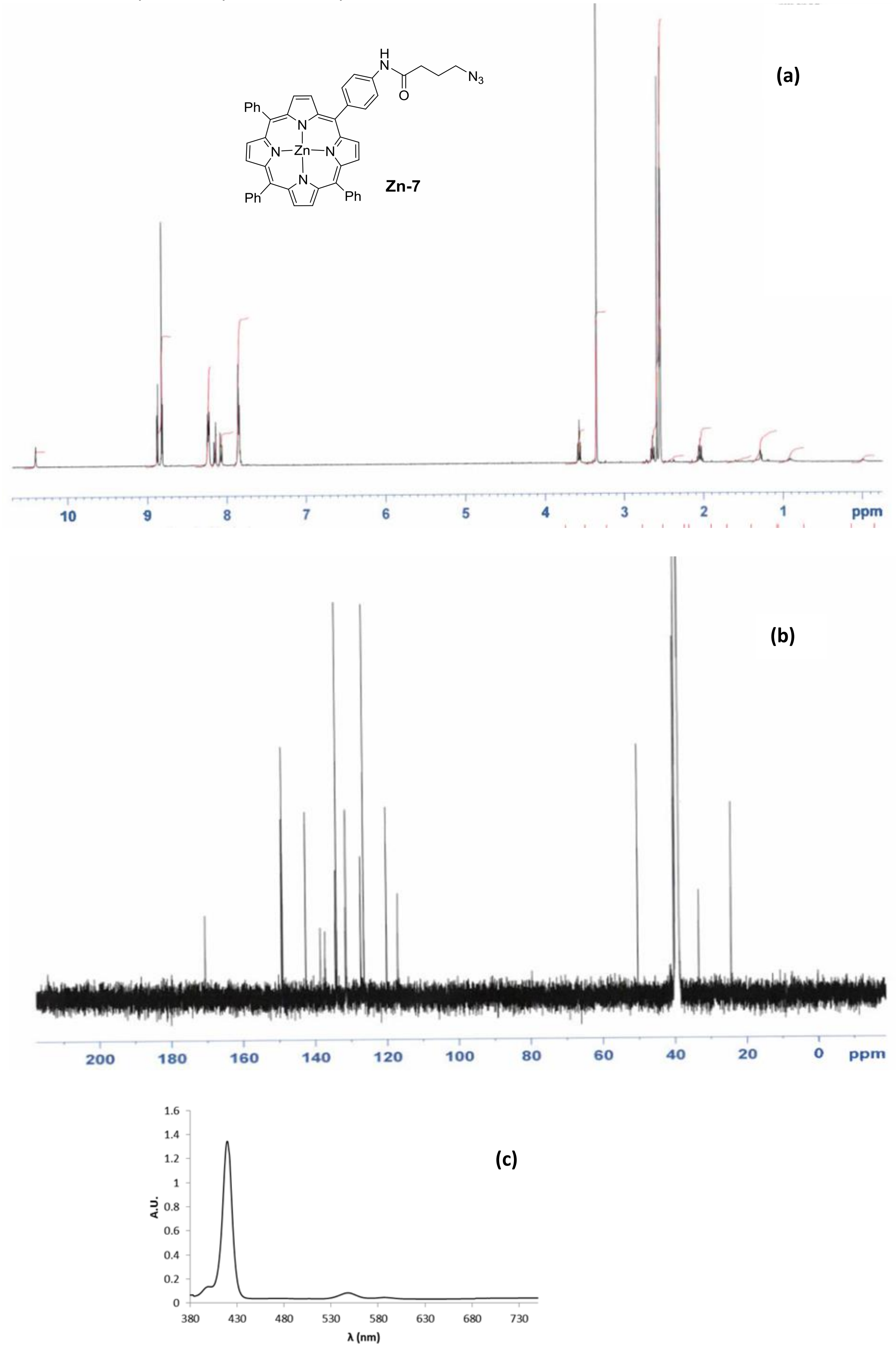

Figure 4 (a)-(b). ^{1}H NMR and ^{13}C NMR spectra in $(CD_3)_2SO$ of **Zn-7** **(c)** UV-visible spectrum in $CHCl_3$ of **Zn-7**.

Electronic Supplementary Material (ESI) for Organic & Biomolecular Chemistry.
This journal is © The Royal Society of Chemistry 2016

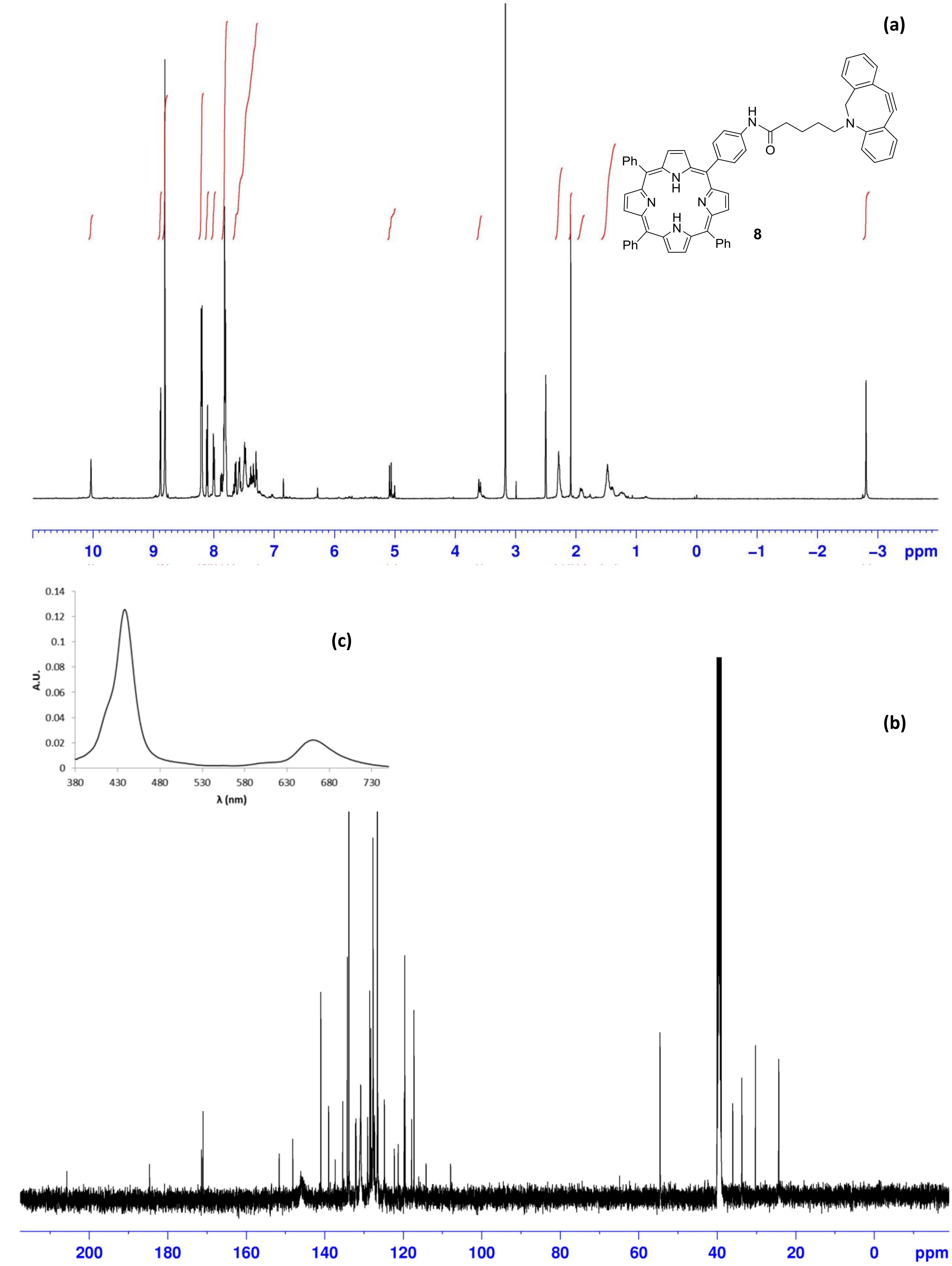

Figure 5 (a)-(b). ^{1}H NMR and ^{13}C NMR spectra in $(CD_3)_2SO$ of **8** **(c)** UV-visible spectrum in $CHCl_3$ of **8**.

This journal is © The Royal Society of Chemistry 2016

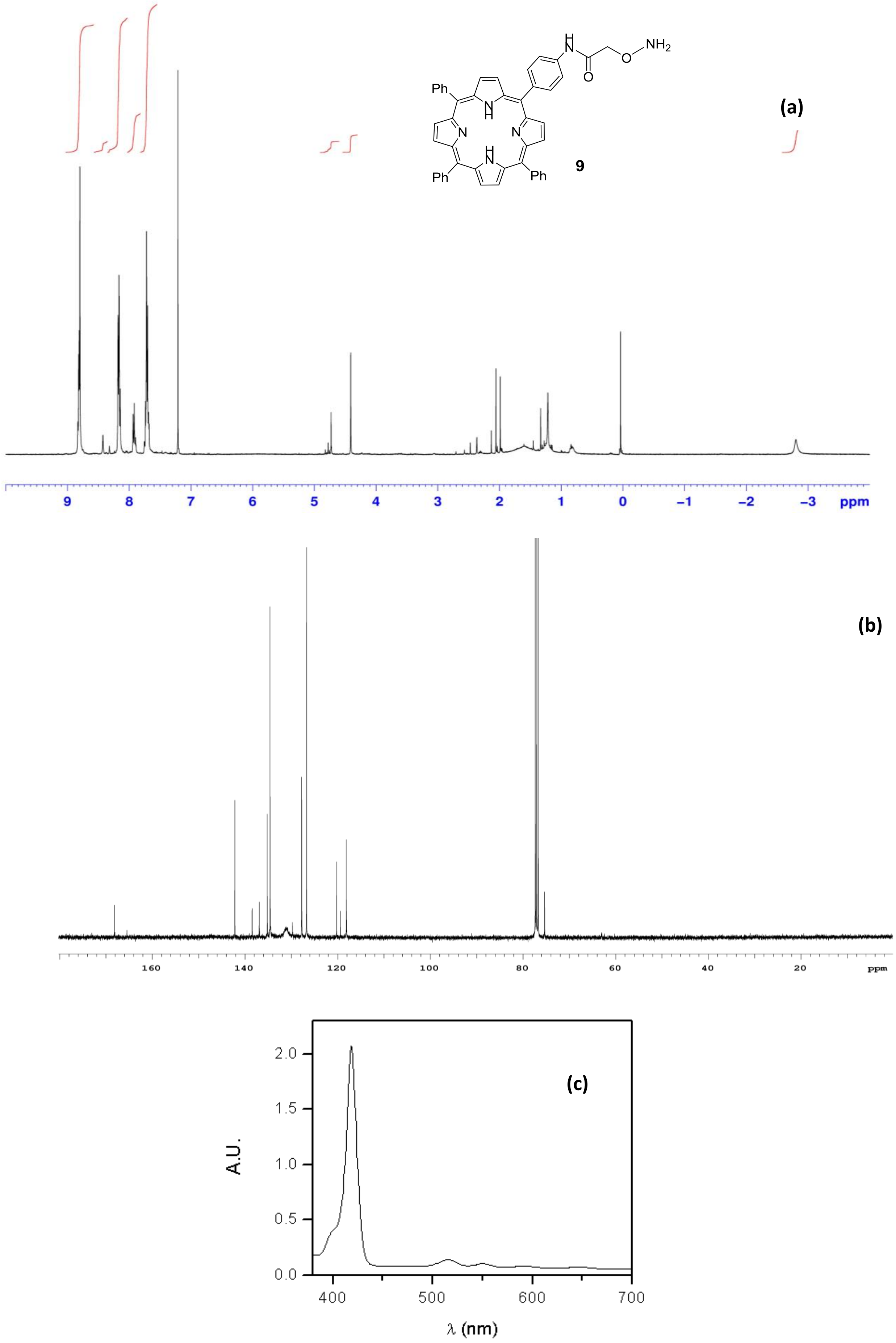

Figure 6 (a)-(b). ^{1}H NMR and ^{13}C NMR spectra in $CDCl_3$ of **9** **(c)** UV-visible spectrum in $CHCl_3$ of **9**.

This journal is © The Royal Society of Chemistry 2016

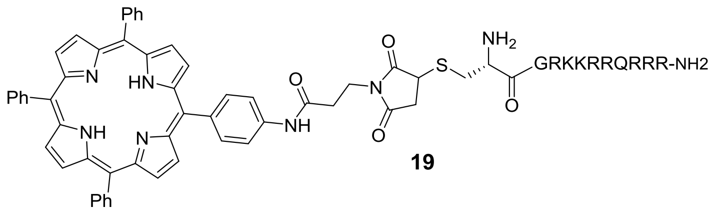

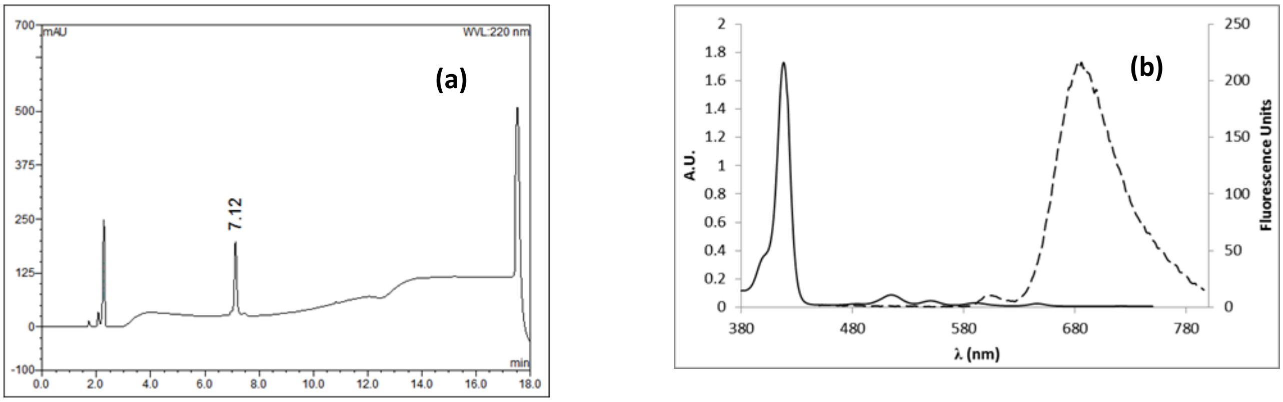

Figure 7 (a) HPLC profile of conjugate **19** (elution Method 2, see General Information); **(b**) UV-visible (solid line) and fluorescence (dotted line) spectra in 0.1% aq TFA of conjugate **19**.

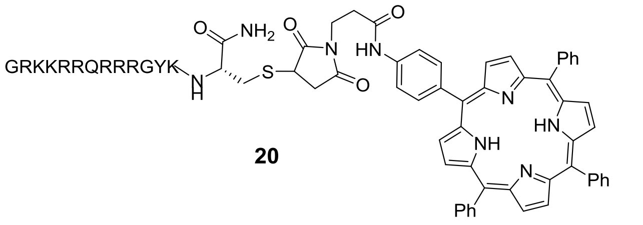

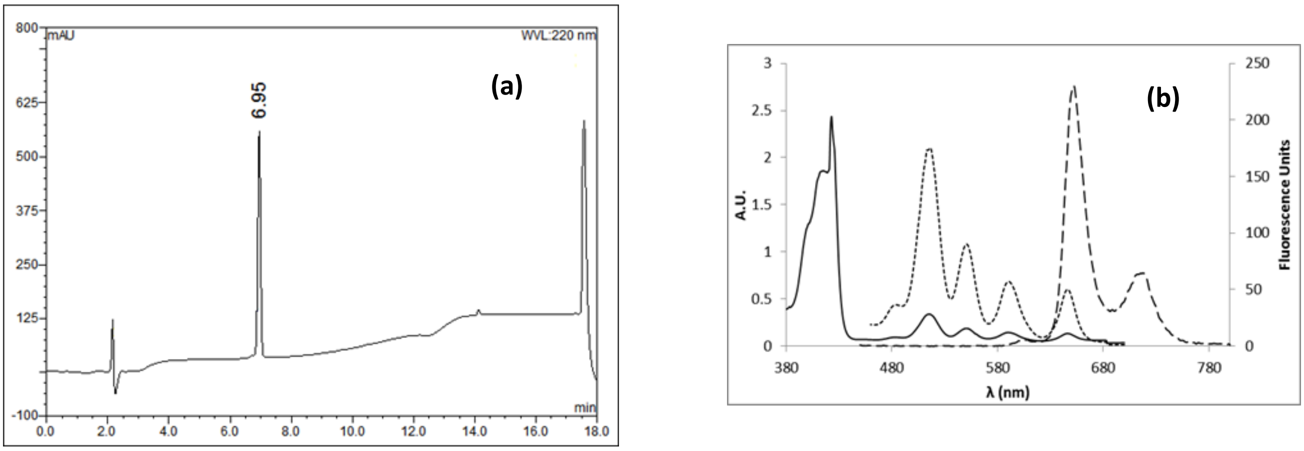

Figure 8 (a) HPLC profile of conjugate **20** (elution Method 2, see General Information); **(b**) UV-visible (solid line) and fluorescence (dotted line) spectra in 0.1% aq TFA of conjugate **20**.

This journal is © The Royal Society of Chemistry 2016

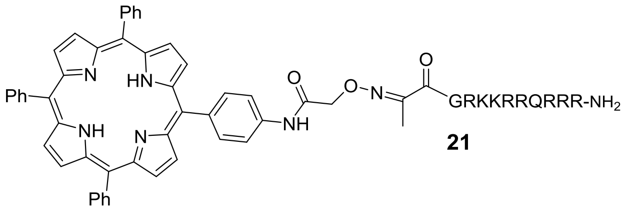

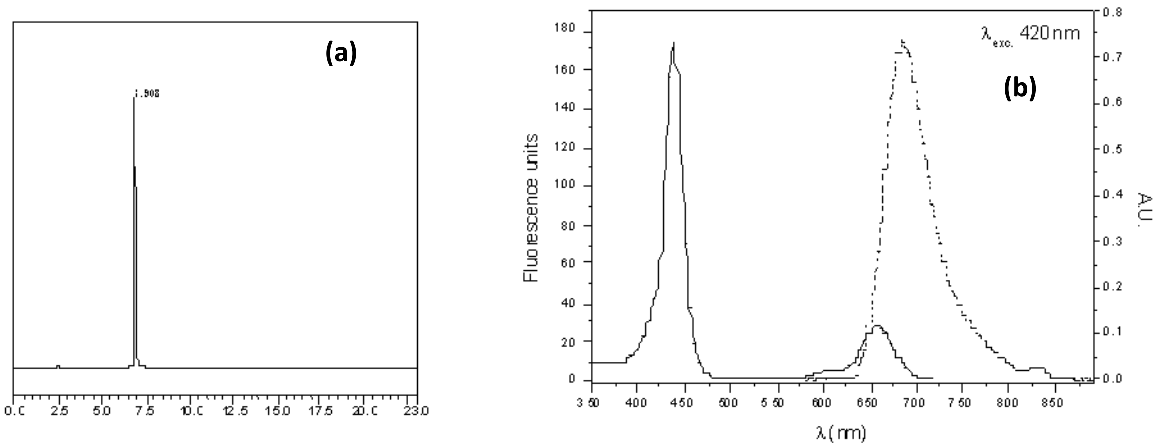

Figure 9 (a) HPLC profile of conjugate **21** (elution Method 2, see General Information); **(b)** UV-visible (solid line) and fluorescence (dotted line) spectra in 0.1% aq TFA of conjugate **21**.

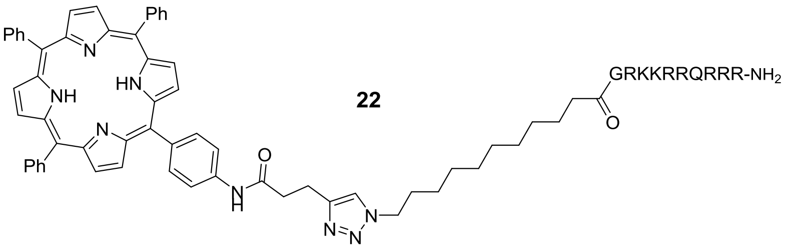

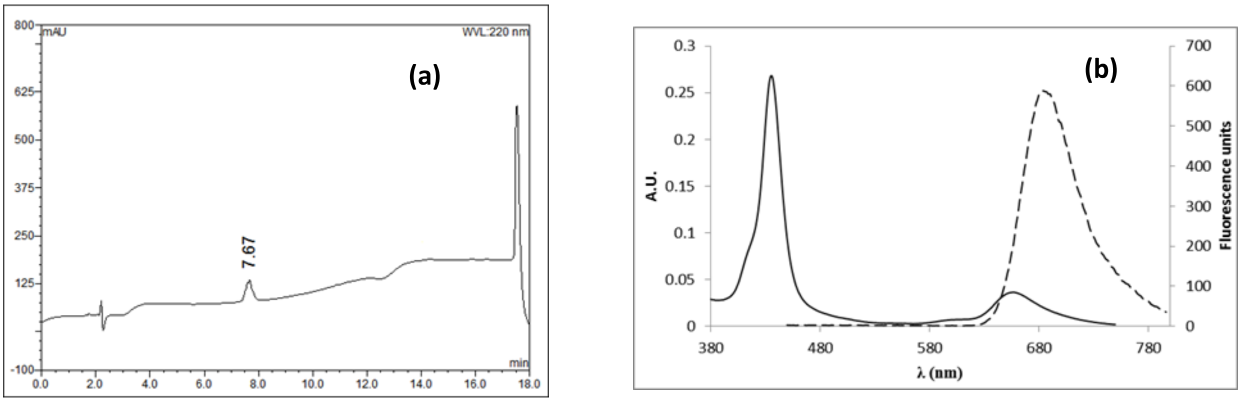

Figure 10 (a) HPLC profile of conjugate **22** (elution Method 2, see General Information); **(b)** UV-visible (solid line) and fluorescence (dotted line) spectra in 0.1% aq TFA of conjugate **22**.

This journal is © The Royal Society of Chemistry 2016

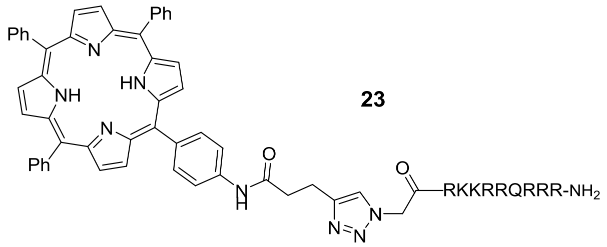

23

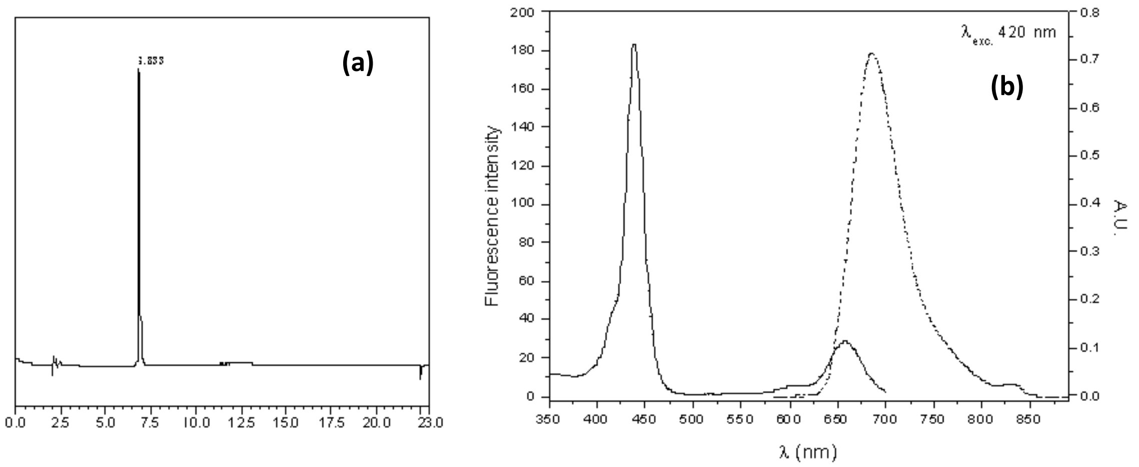

Figure 11 (a) HPLC profile of conjugate **23** (elution Method 2, see General Information); (**b**) UV-visible (solid line) and fluorescence (dotted line) spectra in 0.1% aq TFA of conjugate **23**.

This journal is © The Royal Society of Chemistry 2016

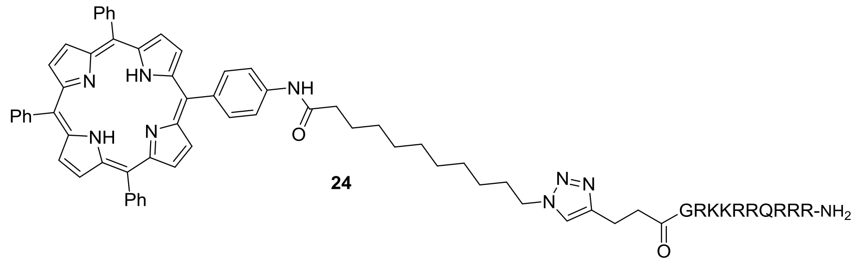

24

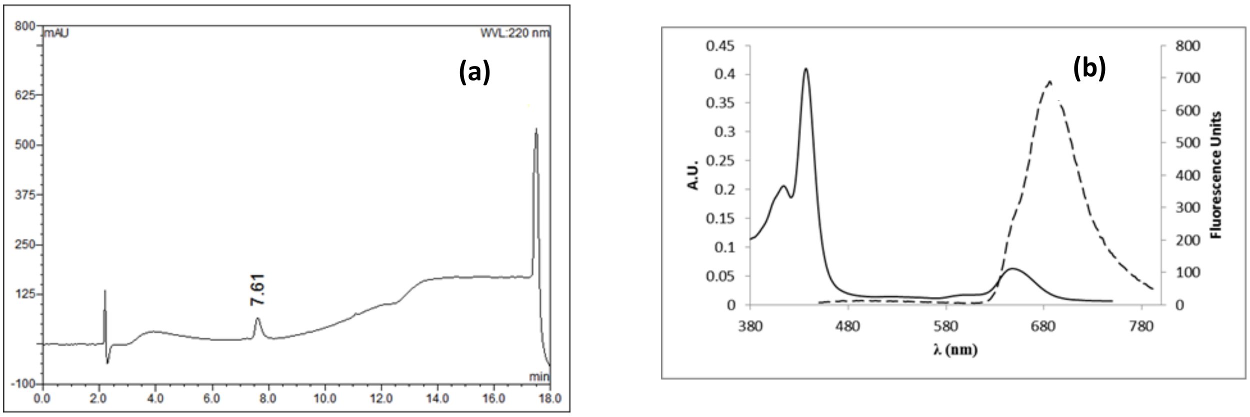

Figure 12 (a) HPLC profile of conjugate **24** (elution Method 2, see General Information); (**b**) UV-visible (solid line) and fluorescence (dotted line) spectra in 0.1% aq TFA of conjugate **24**.

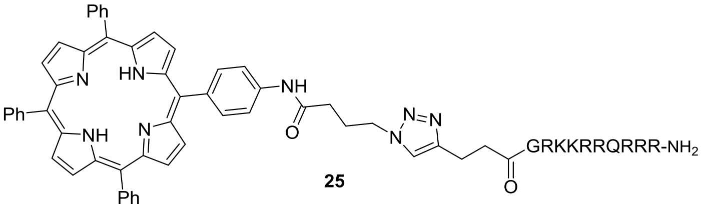

25

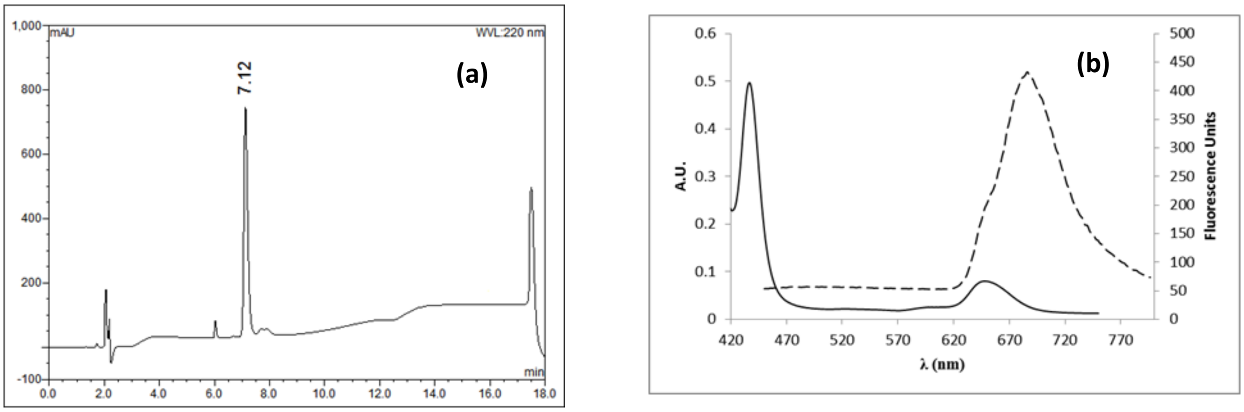

Figure 13 (a) HPLC profile of conjugate **25** (elution Method 2, see General Information); (**b**) UV-visible (solid line) and fluorescence (dotted line) spectra in 0.1% aq TFA of conjugate **25**.

This journal is © The Royal Society of Chemistry 2016

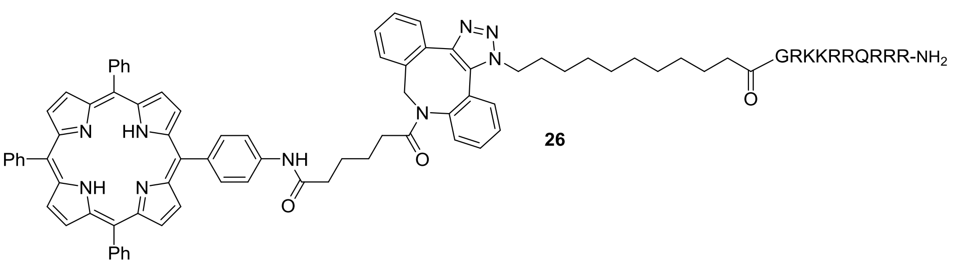

26

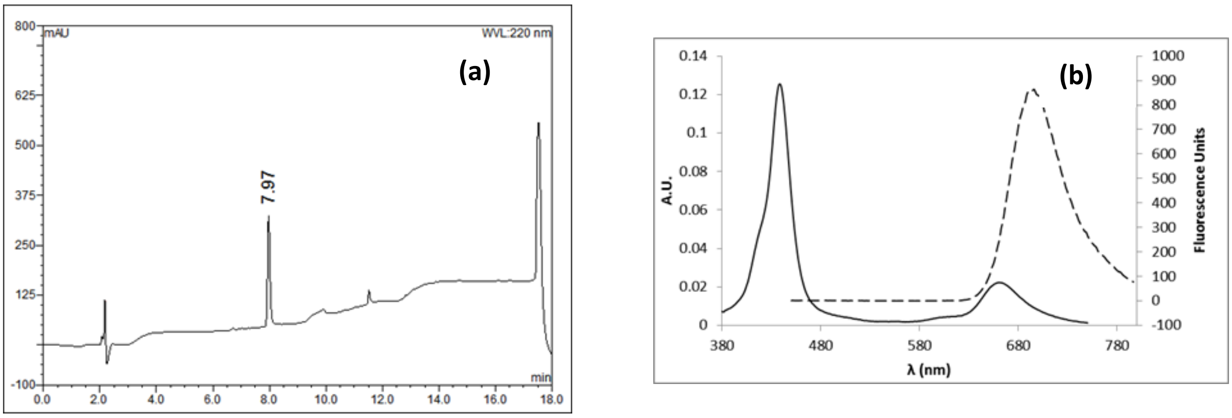

Figure 14 (a) HPLC profile of conjugate **26** (elution Method 2, see General Information); (**b**) UV-visible (solid line) and fluorescence (dotted line) spectra in 0.1% aq TFA of conjugate **26**.

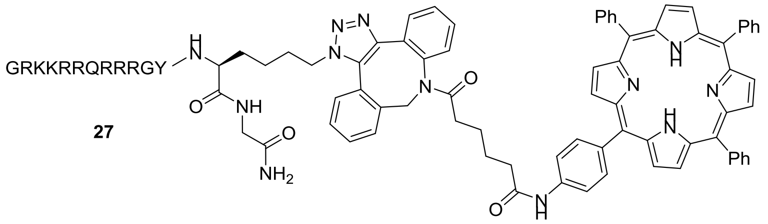

27

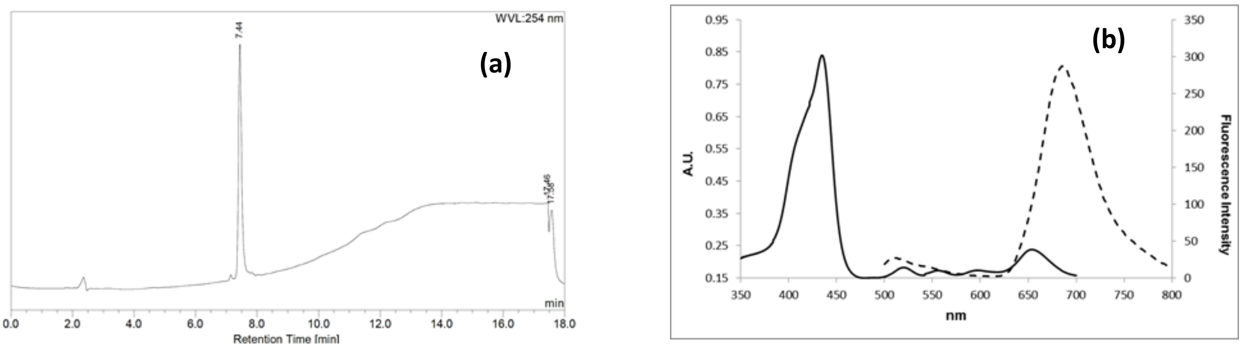

Figure 15 (a) HPLC profile of conjugate **27** (elution Method 2, see General Information); (**b**) UV-visible (solid line) and fluorescence (dotted line) spectra in 0.1% aq TFA of conjugate **27**.

Electronic Supplementary Material (ESI) for Organic & Biomolecular Chemistry.
This journal is © The Royal Society of Chemistry 2016

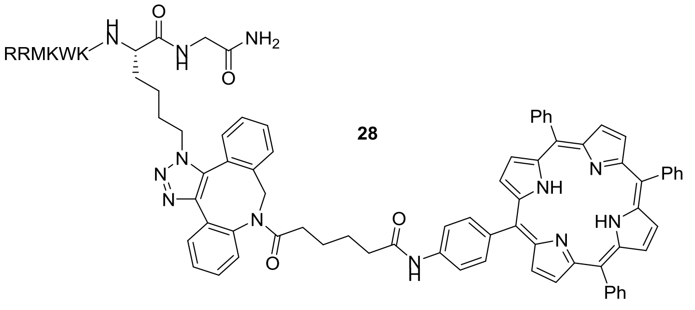

28

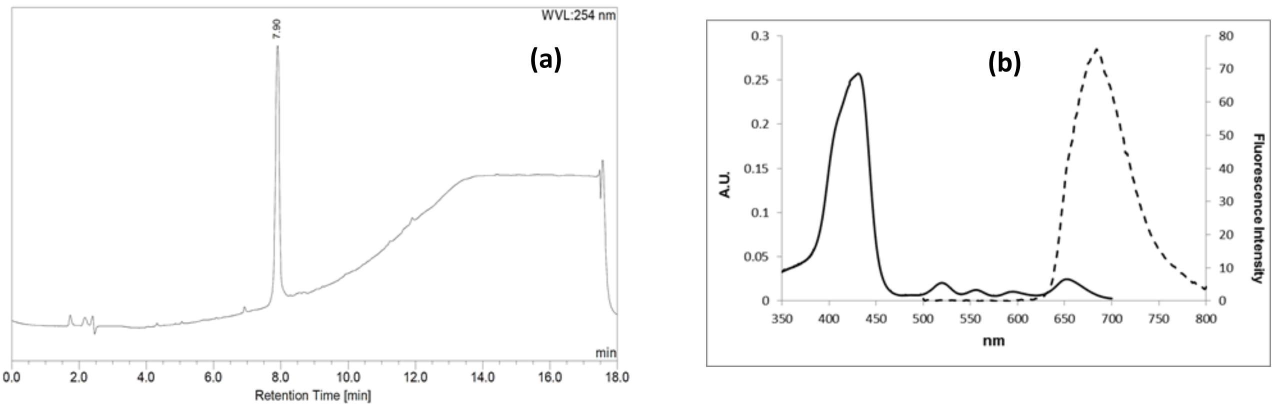

Figure 16 (a) HPLC profile of conjugate **28** (elution Method 2, see General Information); **(b)** UV-visible (solid line) and fluorescence (dotted line) spectra in 0.1% aq TFA of conjugate **28**.

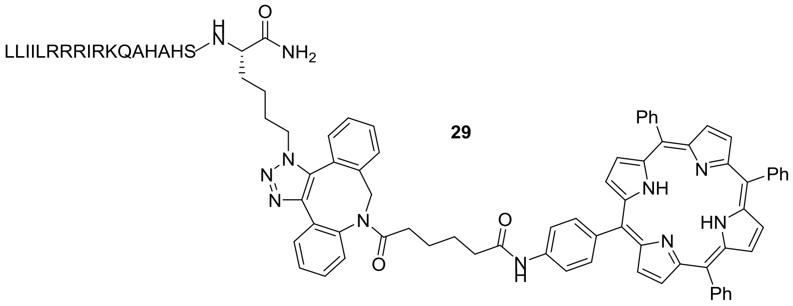

29

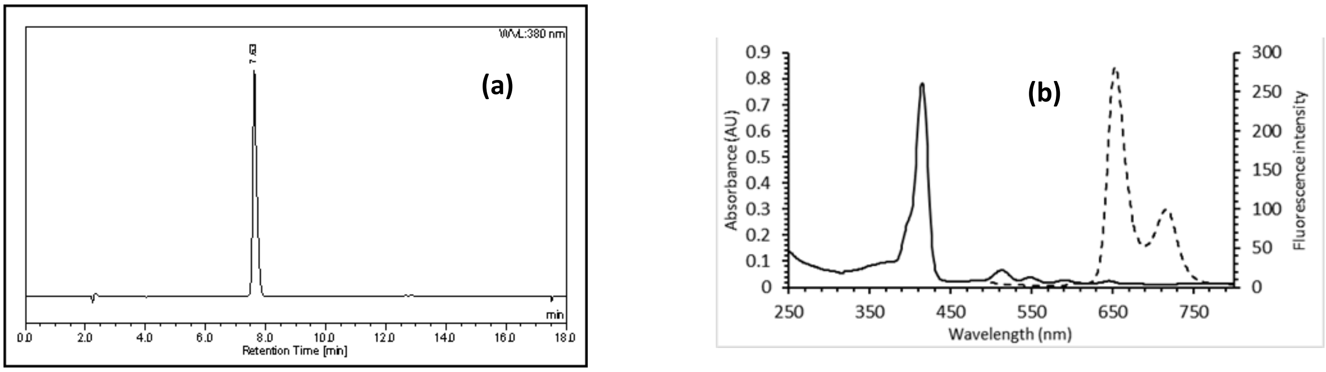

Figure 17 (a) HPLC profile of conjugate **29** (elution Method 2, see General Information); **(b)** UV-visible (solid line) and fluorescence (dotted line) spectra in 0.1% aq TFA of conjugate **29**.
